# Landscapes and mechanisms of CD8^+^ T cell exhaustion in gastrointestinal cancer

**DOI:** 10.3389/fimmu.2023.1149622

**Published:** 2023-04-25

**Authors:** Jia-Tong Ding, Kang-Ping Yang, Hao-Nan Zhou, Ying-Feng Huang, Hui Li, Zhen Zong

**Affiliations:** ^1^ Department of Gastrointestinal Surgery, The Second Affiliated Hospital of Nanchang University, Nanchang, China; ^2^ The Second Clinical Medicine School, Nanchang University, Nanchang, China; ^3^ Queen Mary School, Nanchang University, Nanchang, China; ^4^ Department of Rheumatology and Immunology, The First Affiliated Hospital of Nanchang University, Nanchang, China

**Keywords:** T cell exhaustion, gastric cancer, colorectal cancer, immune checkpoints, immunotherapy

## Abstract

CD8^+^ T cells, a cytotoxic T lymphocyte, are a key component of the tumor immune system, but they enter a hyporeactive T cell state in long-term chronic inflammation, and how to rescue this depleted state is a key direction of research. Current studies on CD8^+^ T cell exhaustion have found that the mechanisms responsible for their heterogeneity and differential kinetics may be closely related to transcription factors and epigenetic regulation, which may serve as biomarkers and potential immunotherapeutic targets to guide treatment. Although the importance of T cell exhaustion in tumor immunotherapy cannot be overstated, studies have pointed out that gastric cancer tissues have a better anti-tumor T cell composition compared to other cancer tissues, which may indicate that gastrointestinal cancers have more promising prospects for the development of precision-targeted immunotherapy. Therefore, the present study will focus on the mechanisms involved in the development of CD8^+^ T cell exhaustion, and then review the landscapes and mechanisms of T cell exhaustion in gastrointestinal cancer as well as clinical applications, which will provide a clear vision for the development of future immunotherapies.

## 1 Introduction

T cell exhaustion (TEX) is an effective low-response T cell state during chronic infection. This hyporeactive state is thought to be due to the inability of pathogens to be rapidly cleared in chronic inflammation and the continued stimulation of this specific T cell proliferation, resulting in the upregulation of many immune checkpoints on the surface of T cells, leading to a reduction in their proliferation and ability to capture pathogens (1, 2). Many studies have been devoted to exploring the mechanisms behind TEX, starting with attempts to overcome its problems. Researchers have tried to explore how TEX causes a reduction in T cell effector function and proliferation levels, as well as leading to the expression of suppressive immune checkpoint receptors and ultimately immune escape of the tumor (3, 4). There is evidence that CD8^+^ T cell infiltration below 2.2% predicts a fourfold higher risk of disease progression after cancer surgery (hazard ratio (HR) = 3.84, p < 0.01), but the proportion of CD8^+^ T cells does not correlate with other clinical data, suggesting that the mechanisms that trigger differences in T cell heterogeneity and kinetics are unclear (5, 6). Therefore, it is particularly important to identify the molecular determinants that regulate the number, spatial distribution, and heterogeneity of CD8^+^ T cells in tumors, which could help more patients to benefit from immunotherapy (7).

Current studies on TEX have found that the mechanisms behind it may be closely related to transcription factors and epigenetic regulation (8, 9). For example, in a study using a mouse model of chronic LCMV infection, the transcription factor TCF-1 was found to promote the function of depleted CD8^+^ T cells by promoting the expression of a series of key effector function-related transcriptional regulators, including Foxo1, Zeb2, Id3, and Eomes (10). A study identified progressive heritable methylation programs that limit T cell expansion and clonal diversity during PD-1 blockade therapy, and when these programs were reversed, TEX no longer inhibited immune checkpoint blocker (ICB) therapy (11). These findings suggest that transcription factors and epigenetic regulation may serve as biomarkers of CD8^+^ TEX and potential immunotherapeutic targets, which would provide precision therapy for tumor immunization patients (12).

The importance of TEX in tumor immunotherapy has been confirmed by many studies, however, what needs more attention is that gastrointestinal tumors are perhaps more suitable for deeper work in this direction than other tumors. For example, a pan-cancer analysis showed that the presence of a lower percentage of triminal CD8^+^ Tex and a higher percentage of CD8+ Trm cells in STAD tissues implies that the gastric cancer tumor microenvironment has a better composition of anti-tumor T cells, which would point to the development of more promising precision-targeted immunotherapies (3, 6, 9). There are many ongoing studies on CD8^+^ TEX in gastrointestinal cancers regarding immune checkpoint inhibition, CAR T cells, and synergistic therapy with chemotherapy, but many mechanisms are still unclear (13).

Therefore, this review will describe the origin, function, detection modalities, and mechanisms involved in CD8^+^ TEX, and review TEX in gastrointestinal cancers and related clinical applications, which will provide a clear and comprehensive vision for the development of future immunotherapies.

## 2 CD8^+^ T cell exhaustion

### 2.1 Origin and function of CD8^+^ T cell

CD8^+^ T cells are cytotoxic T lymphocytes (CTL cells) that are produced by the body to fight against viruses, tumors, and other pathogens (14). They produce and express αβ-T cell receptors with CD8 in the thymus and destroy them by recognizing MHC class I on target cells (15). When the body’s CD8^+^ T cells or function is diminished, anti-tumor immune function will decrease and the risk of tumor growth and cancer metastasis will increase (16).

**Figure 1:**
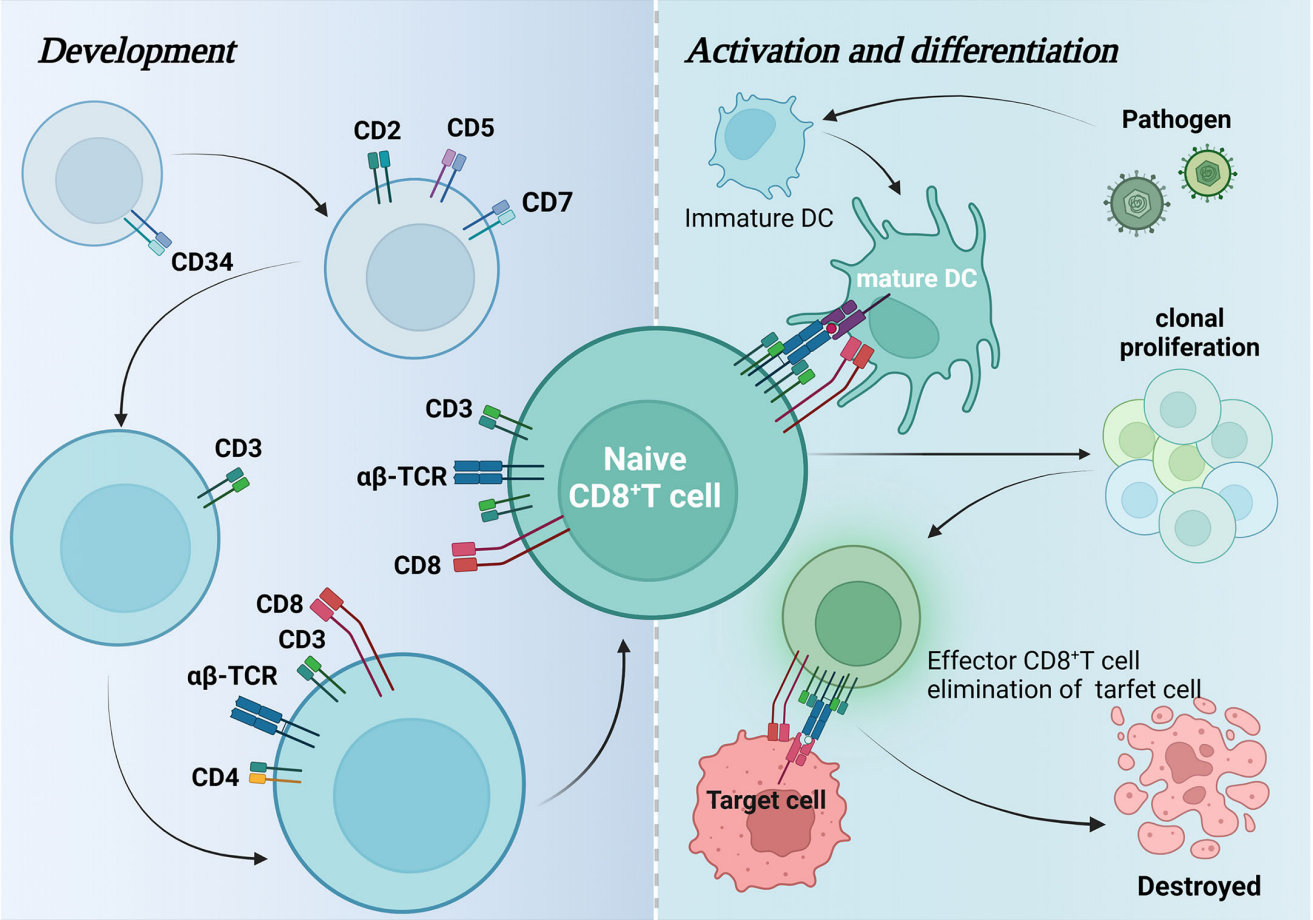
Development, activation, and differentiation of CD8^+^ T cells. This figure maps the changes in surface-specific antigens during CD8^+^ T cell development and how they are activated by dendritic cells to differentiate and generate effector CD8^+^ T cells that attack pathogenic target cells and eventually cause them to autophagy.

CD8^+^ T cells develop from CD34^+^ hematopoietic stem cells located in the bone marrow, which express CD2, CD5, and CD7 before leaving the bone marrow and entering the thymus to become CD3-expressing lymphoid progenitors, and subsequently undergo a double negative (DN) (CD8-CD4-) phase and a double positive (DP) phase (CD8^+^ CD4^+^) and finally became single positive (SP) CD8^+^ thymocytes. These cells are selected by positive and negative clones to become CD8^+^ T-αβ cells and are released into the circulation (5, 17). Antigen-presenting cells such as dendritic cells (DCs) usually present endogenous antigenic peptides in MHC class I molecules (18). CD8^+^ T cells are activated by recognition of antigenic peptides by CD8^+^ co-receptors on TCRs and CD8^+^ T cells and activated CD8^+^ T cells can lead to clonal expansion of antigen-specific CD8^+^ T cells, which then differentiate into effector or memory cells(**Figure 1**) (19).

CD8^+^ T cells can exert antiviral or antitumor immune effects by mediating direct or indirect killing responses to target cells (20, 21). The main pathways of killing are: a) causing apoptosis of target cells through the release of lysozyme in intercellular contacts; b) acting on the target cell-expressed receptor Fas *via* Fas ligand (Fas-L), leading to apoptosis of target cells through a cysteine-dependent pathway; and c) indirectly inducing death of peripheral tumor cells through the secretion of cytokines (22, 23).

### 2.2 Definition and mechanism of CD8^+^ TEX

CD8^+^ TEX has been used to describe the unresponsive, low-effect state of T cells during tumor progression and is commonly seen in advanced progressive tumors that exhibit a host-cancer cell stalemate pattern (12, 24, 25). In the early stages of tumor development, i.e., before sufficient numbers of cancer cells and tumor antigens form and/or appear, tumor-reactive CD8^+^ T cells may remain in a state of ignorance (26, 27). As cancer progresses, CD8^+^ T cells are continuously stimulated by tumor antigens and enter a late dysfunctional state late (28).Exhausted-like T cells isolated in progressive tumors will exhibit tumor-infiltrating lymphocytes (TIL) that are responsive to tumor antigens expressing multiple inhibitory receptors and failing to produce effector cytokines or cytotoxic molecules (29–32). The mechanism that CD8 +T cells cannot eliminate cancer is attributed to factors such as cancer cells and TME, etc. (33–35).

Mechanisms mediated by cancer cells include loss of MHC expression, loss of antigen, loss or defect in antigen presentation, or expression of inhibitory receptor ligands. Loss of MHC class I expression of PD-L1 or other ICPs has been noted in many cancer types (36). expression of most components of the MHC I antigen presentation pathway is nonessential for cell survival and therefore is usually loss of function in cancer cells, leading to T cell depletion. transcription factors such as NLRC5, IRF1 and IRF2 are thought to play an important role in induced MHC I transcription expression and do not affect cell viability (37, 38). A new study indicates that post-translational protein modification, SUMOylation, also induces cancer cells to evade CD8^+^ T cell-mediated immune surveillance by inhibiting MHC-I antigen processing and presentation mechanisms (39).

Mechanisms mediated by TME include TGFβ, IL-10, and metabolic interaction. Tumor microenvironment (TME) is an ecological niche that can suppress CD8^+^ T cell immunometabolism and cytotoxicity. In a study of malignant breast cancer cells, tumor-derived extracellular vesicles could transport active TGF-β type II receptors (TβRII) to CD8^+^ T cells, thereby activating SMAD3 expression in CD8 T cells, ultimately leading to TEX and resulting in failure of immunotherapy (40). IL-10 release from HMOX1 myeloid cells induces TEX in the glioblastoma microenvironment (41). Cetuximab-based IL-10 fusion protein has also been shown to prevent dendritic cell-mediated TEX by regulating the IFN-γ pathway (42). Notably the role of lactate metabolism in the regulation of TEX is controversial. It has been suggested that lactate increases the stemness of CD8^+^ T cells and enhances antitumor immunity (43), while it has been suggested that tumor-derived lactate inhibits the cytotoxicity of CD8^+^ T cells (44, 45). Therefore, finding a level of lactate that balances cytotoxic CD8^+^ T cells with enhanced antitumor immunity is worth exploring. The mechanisms of T-cell loss are also influenced by tumor, treatment modality, and model, and research in this area is scarce yet necessary.

### 2.3 Detection of CD8^+^ TEX

Single-cell transcriptomics is an important tool used to study the mechanisms of CD8^+^ TEX in cancer (46). For example, one study investigated the heterogeneity of high-grade serous ovarian cancer within its TME by scRNA-seq and found high expression of the immunosuppressive receptor TIGIT on CD8+ TEX cells. In a mouse model, the study blocked TIGIT, leading to f slowing of ovarian cancer tumor growth (47).These studies could provide new perspectives for further immunological studies and immunotherapy of relevant tumors (47, 48).

TEX as a sign-specific cell type needs to be distinguished from canonical functional T cell subpopulations phenotypically similar to it [e.g., naïve T cells (TN), effector T cells (TEFF), and memory T cells (TEME), often with great difficulty, despite the great functional, transcriptomic, and epigenomic differences in TEX (49, 50). This is because TEX expresses many markers that are equally expressed by TEFF and TEME (51). However, TEX has significant heterogeneity, such as TEX subpopulations with ancestral-progenitor relationships, or groups with different degrees of failure or homeostatic potential, which may be directly associated with disease progression. And TEX has a guiding role in the use of clinical immunosuppressants, which can be used to determine the suitability of immunosuppressant use and to predict the prognosis (32). Therefore, considering TEX heterogeneity, many studies have used mass spectrometry flow techniques (CyTOF) to perform high-dimensional number analysis for accurate assessment of TEX [47].

Although flow cytometry and single-cell transcriptomics have provided unprecedented insightful solutions to reveal the mechanisms controlling CD8^+^ T cell function in cancer, however, these techniques have failed to capture important spatial-spatial information, including intercellular interactions and tissue localization. Many new studies have now identified intra-tumor immune ecological sites, tertiary lymphoid structures (52), and tumor-draining lymph nodes as key sites of intercellular communication (53), and regulation of these sites plays an important role in the spatial determination of CD8^+^ T cell function.

## 3 Mechanism of CD8^+^ TEX

During acute infection, naïve CD8^+^ T cells are activated and differentiate into effector CD8^+^ T cells, including short-lived effector cells (SLEC) and memory precursor effector cells (MPEC) (54–56). Effector CD8^+^ T cells are characterized by extensive reprogramming of transcriptional network, epigenetic modulation, and metabolism, and the acquisition of cardinal effector function, including changed tissue homing, and dramatic numerical expansion (54, 55). After the clearance of antigens, the majority of effector T cells die, and the subset-MPEC would then convert into memory CD8^+^ T cells. Memory CD8^+^ T cells could be reactivated easily and rapidly when encountering the secondary infection, and their maintenance is independent of antigen stimulation but a stem cell-like self-renewal dependent on interleukin-7(IL-7) and IL-15 (54). Whereas during chronic infection or cancers, an altered differentiated state of T cells appears and is termed TEX due to persistent antigen stimulation. Exhausted CD8^+^ T cells have various cardinal features, including progressive loss of effector function, sustained upregulation of multiple co-inhibitory receptors such as PD-1, CTLA4, LAG3, and TIM3, downregulation of co-stimulatory receptors, and altered transcriptional network, epigenetic modulation, and metabolism compared to functional effector cells and memory T cells (57). Contrary to memory T cells, exhausted CD8^+^ T cells respond poorly to IL-7, and IL-15, and their persistence are *via* continuous stimulation of antigens (58–60), although a minority of them might be persistent without the continuous antigen stimulation in some contexts (61–63). Of note, while exhausted T cells are relatively hypofunctional, they still provide a crucial killing effect against pathogens, which exerts inseparable roles for host-pathogen balance, hence, another thought toward TEX has been popular recently that the generation of TEX is a protection mechanism for hosts to avoid the inflammation damage due to immune system hyper-activated by persistent antigen stimulation. Whereas the generation of TEX involves multiple factors, herein, we manage to review the correlated studies to describe the mechanism of TEX from the perspective of transcription factors and epigenetic modulation.

### 3.1 Transcriptional factors

It has been known that transcription factor T-bet and Eomes are both essential for the differentiation of effector CD8^+^ T cells and memory CD8^+^ T cells during acute infection, where their roles are partially overlapping but the expression discrepancies also exist in effector and memory CD8^+^ T cells, indicating that they respectively have unique function in the development process of effector CD8^+^ T cells and memory CD8^+^ T cells (55). In brief, early effector CD8^+^ T cells (T-bet^+^Eomes^+^) increase the expression of T-bet to turn into terminally differentiated effectors (T-bet^++^Eomes^+^), while memory CD8^+^ T cells express a higher level of Eomes (T-bet^+^Eomes^++^) (64). However, if the acute infection progresses into a chronic infection, terminally differentiated effectors would reduce the expression level of T-bet but express more Eomes (T-bet^+^Eomes^+++^) to execute the exhaustion program, instead of differentiating into memory CD8^+^ T cells. T-bet and Eomes influenced the fate decision between memory T cells and exhausted T cells. Exhausted CD8^+^ cells expressed less T-bet and more Eomes versus CD8^+^effectors, and higher Eomes expression seemed to predispose CD8^+^ effectors to become exhausted T cells (T-bet^+^Eomes^+++^) rather than memory T cells (T-bet^+^Eomes^++^) when encountering persistent infection. During chronic LCMV infection, the expression pattern of T-bet and Eomes is also closely associated with the differentiation within the heterogenous Exhausted CD8^+^ T cells, and it seems that T-bet and Eomes have antagonistic roles for Tex development (65). Researchers found that two Tex subsets identified according to the expression levels of T-bet and Eomes, in conjunction with PD-1, have different residual effector functions although both subsets were effector impaired versus memory CD8^+^ T cells. T-bet^Hi^Eomes^Lo^PD-1^int^ Tex cells could still proliferate and release medium amounts of TNFα and IFNγ, and they were potential to be reinvigorated with anti-PD-1/PD-L1 therapy (66). Whereas T-bet^Lo^Eomes^Hi^PD-1^Hi^ Tex cells were less potential to proliferate, produced lower quantities of effector cytokines, and simultaneously expressed higher levels of other inhibitory receptors except for PD-1 and CTLA4, including CD160, Lag-3, and Tim-3. It suggested that a high expression level of T-bet was correlated to a progenitor-like Tex subset with mild exhaustion performance, high expression of Eomes was associated with a Tex subset with severe exhaustion manifestation, and these two transcription factors have an opposite regulatory effect on the expression of Tex markers including PD-1. Although exactly how they influenced the fate decision between memory T cells and exhausted T cells and the development process of Tex remains poorly understood, more comprehensive events that appear between T-bet and Eomes and exhaustion program have recently been revealed by Mclane et al., which exhibited that T-bet and Eomes competed for the same binding DNA sequences, including the *Pdcd1* (encoding for PD1) (67). A high level of nuclear T-bet vigorously inhibited Pdcd1 transcription, while a low level of nuclear T-bet gave rise to Eomes-mediated weaker repression on *Pdcd1* in Tex, and blocking PD-1 signaling in Tex could upregulate T-bet level, which restored potent inhibition for Pdcd1 transcription and T-bet associated gene expression of activation, chemotaxis and homing (67).

In 2016, He et al. found that during chronic infection, a subset of exhausted CD8^+^ T cells expressing the chemokine receptor CXCR5 could migrate into B-cell follicles, which was similar to follicular helper T cells, and the CXCR5^+^ subset expressed lower amounts of inhibitory receptors and retained more powerful cytotoxicity compared with CXCR5^-^ subset (68). This specific CXCR^+^ subset was also observed in HIV patients, and its amount was inversely associated with viral load. Besides, when adoptively transferred into chronically infected mice, the CXCR^+^ subset exhibited a synergistic effect with the anti-PD-L1 treatment to reduce viral load versus CXCR^-^ subset, which showed a greater therapeutic potential (64). In succession, another study also reported that in the mice model with chronic infection of LCMV, the CXCR5^+^PD-1^+^ subset expressed more co-stimulatory molecules including ICOS and CD28 versus the CXCR5^-^PD-1^+^ subset, and the specific subset was able to proliferate, almost accounting for the whole proliferative burst, in response to PD-1 signaling blockade (64). Besides, the transcription profiling demonstrated that the CXCR5^+^PD-1^+^ subset was highly enriched for the transcription factor TCF-1, indicating an important role of TCF-1 in the progenitor-like Tex subset (64). Later, multiple studies demonstrated that the TCF-1^+^ subset represents the progenitors of exhausted T cells, which proliferates more TCF-1^+^ progenitors, or loses the expression of TCF-1 and differentiates into the terminally effector subset (32, 69–71). On the one hand, TCF-1 itself functions as a direct downstream effector of the WNT/β-catenin signaling pathway that exerts essential roles for stem cell self-renewal, on the other hand, the depletion of TCF1 indeed could deprive the proliferative potential and aggravate the exhausted manifestation of Tex cells in chronic infection or tumors (72–74). Hence, people gradually recognized that TCF-1 seems to take up a core position to regulate various transcription factors and further edit the exhaustion program. Transcriptional profiling suggested that TCF-1 cKO CD8^+^ T cells exhibited increased Tim^high^Blimp^high^ gene expression signatures but reduced Tim^low^Blimp^low^ gene expression signatures, indicating that TCF1 promoted the downregulation of Tim and Blimp, and expressed lower amounts of mRNA for transcription factors, including *Bcl6*, *Ikzf2*, and *Aff3*, and surface receptors such as *Ccr7*, *CXCR5*, *Il23r*, *Tnfsf8* (encoding CD30L) and *Sell* (encoding CD62L) compared to WT CD8^+^ T cells (68). Microassays showed that TCF1 down-regulated *Prdm1*(Blimp1) and *Havcr2* (Tim3), which might implicate the repression mechanism for Blimp1 and Tim3 (71). On the contrary, Bcl6, a crucial regulator of follicular helper T cell differentiation that counteracts Blimp1 activity (75, 76), was found to be induced by overexpression of TCF1 (68). Additionally, in the early phase of Tex development, TCF1 repressed the expression of T-bet and ID2 and promoted the expression of Eomes, c-Myb, and Bcl-2, mediating a transcription factor transition from Tex precursors to terminally differentiated Tex (69).

**Figure 2:**
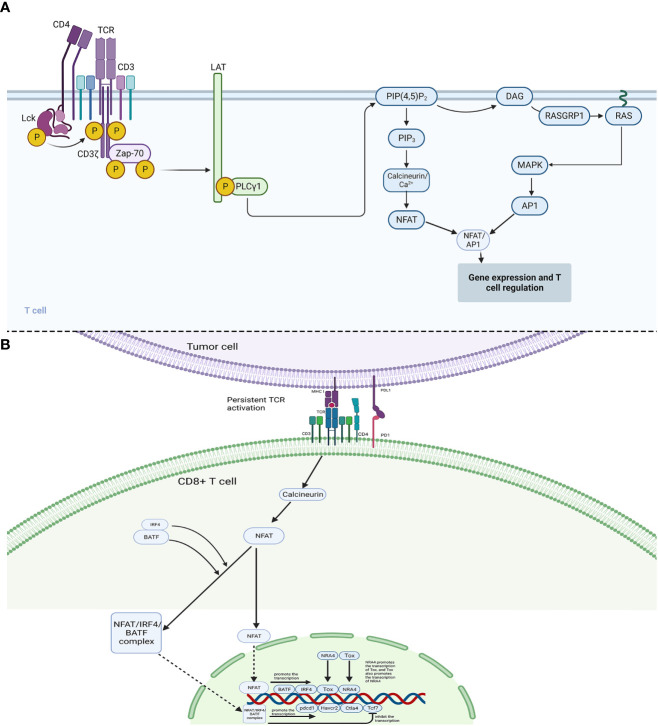
Mechanisms of CD8^+^ T cell regulation and exhaustion. **(A)**: In acute infection, phosphorylated Lck phosphorylates CD3 and ζ chains, which then link ZAP-70. ZAP-70 could phosphorylate LAT, and then LAT would bind PLCγ1, which activates PIP(4,5)P2. Subsequently, in one pathway, PIP(4,5)P2 activates DAG/RAS/MAPK and AP1, in another pathway, PIP(4,5)P2 could induce the activation of Calcineurin/Ca+ and NFAT. **(B)**: In the case between tumor cells and CD8+ T cells, the interaction between PD1 and PDL1 interferes the original powerful immune-activated pathways, which could induce abnormal NFAT signaling to promote Tex. Continuous TCR activation leads to the activation of calcineurin pathways, which dephosphorylates the cytoplasmic nuclear factor of activated T cells (NFAT). The cytoplasmic NFAT would then translocate into the nucleus and promote transcription of BATF, IRF4, Tox, and NRA4. Cytoplasmic BATF and IRF4 proteins could also cooperate with cytoplasmic NFAT protein (NFAT/IRF4/BATF complex), which then translocates into the nucleus. The nuclear NFAT/IRF4/BATF complex could promote the transcription of inhibitory receptor genes, including pdcd1(PD-1), Havcr2(TIM-3), and Ctla4(CTLA-4), and repress the gene Tcf7(TCF-1).

Persistent antigen stimulation is considered necessary for the execution of the exhaustion program, in other words, TCR activation is indispensable for the initial maintenance of exhausted CD8^+^ T cells. Whereas acute infection also requires TCR activation, hence, regarding the outcome discrepancies, a different pattern should exist in TCR activation between acute infection and persistent infection and tumors. In acute infection, activation of TCR and CD28 on the surface of T cells generates signaling cascades, which further activates transcription factor NFAT as well as its partner AP1 to form the NFAT/AP1 complex, and the complex is crucial for the activation of CD8^+^ T cells and the acquisition of various effector functions. Hence, NFAT and AP1 are both elevated during the development of effector cells. In contrast, in exhausted CD8^+^ T cells, NFAT (especially NFAT2) is separately upregulated, while the expression level of AP1 is lower (77), indicating that NFAT might exert its role independent of AP1 in exhausted T cells (**Figure 2**). Consistently, Martinez et al. confirmed that NFAT proteins triggered the alteration of the transcriptional network to execute the exhaustion program, and NFAT that was engineered as sequestered from AP1 protein could strongly induce exhaustion (78), demonstrating that the individual NFAT exerted essential roles in the execution of exhaustion program. Engineered NFAT proteins were also proved to bind to the regulatory sites of inhibitory receptors, including LAG3, PD-1, and TIM3, thus enforcing the exhaustion program (74). Of note, in the study of Martinez et al. (74), the NFAT proteins were designed as sequestered from AP1 protein, and it was uncertain that individual NFAT induced the above outcomes by itself or cooperating with other transcription factors, maybe the modulating axis also existed between NFAT and other molecules. A recent study showed that, in response to persistent TCR signaling, NFAT proteins promoted the expression of IRF4 (interferon regulatory factor 4) and BATF (basic leucine zipper ATF-like transcription factor), and NFAT proteins could cooperate with IRF4 and BATF (NFAT/IRF4/BATF) to induce the expression of genes involved in multiple pathways mediating exhaustion, for example, *Pdcd1*(PD1), *Havcr2*(TIM-3), and *Ctla4*(CTLA-4), whereas the NFAT/IRF4/BATF complex repressed the expression of *Tcf7*(TCF-1) which was necessary for the maintenance of progenitor-like T cells in Tex, suggesting that NFAT/IRF4/BATF complex promote the development of terminally differentiated Tex cells through repressing TCF-1 (79). Additionally, chronic tumor antigen stimulation as well as induced NFAT activation could give rise to the expression of transcription factor Tox that is highly expressed in dysfunctional tumor-specific CD8^+^ T cells and exhausted CD8^+^ T cells during chronic infection (80, 81). Tox is a crucial center regulator of exhaustion. Ectopic expression of Tox in effector T cells induced a transcriptional program associated with TEX *in vitro*, and deletion of Tox could rescind the exhaustion program in tumor-specific CD8^+^ T cells, for example, Tox-deleted exhausted CD8^+^ T cells could not upregulate genes encoding for inhibitory receptors, including *Entpd1*, *Pdcd1*, *Tigit*, *Havcr2* and *Cd244* and reserved high expression level of TCF-1 (37). Similarly, transcription factor NR4A was also confirmed to be induced by NFAT in exhausted T cells, and NR4A-deficient CD8^+^CAR^+^PD-1^high^TIM3^high^ tumor-infiltration T cells exhibited upregulated cytokine expression and reduced expression of inhibitory receptors (82). The authors also proved the existence of positive-feedback regulation in the expression between Tox and NR4A, thus cooperating to promote CD8^+^ TEX (77). Therefore, it suggests that Tox and NR4A are the downstream targets of NFAT and contribute to TEX to a great extent, which might be potential to serve as therapeutic targets for cancer control.

### 3.2 Epigenetic modification

Although terminally differentiated exhausted CD8^+^ T cells express higher amounts of PD-1 receptor compared to initial progenitor exhausted CD8^+^ T cells, anti-PD-1: PD-L1 treatment almost could not reinvigorate the terminally differentiated CD8^+^ subset, suggesting that the exhaustion program is also fixed by other factors. Focused on the limitation of anti-PD-1:PD-L1 treatment, with genome-wide epigenetic profiling technologies, researchers found that Tex acquired a distinct epigenetic landscape compared to effector T cells and memory T cells, and only ~10% of the epigenetic landscape was remodeled after PD-1 blockade, indicating that Tex was a distinct lineage of CD8^+^ T cells and the stable epigenetic landscape of Tex might limit the current immunotherapies (83). Additionally, even in the absence of antigens, the results remained similar (84–86). A recent study revealed that using mice models, after transferring the Tex cells into the infection-free mice, although partial Tex cells that were TCF-1^+^ Tex progenitors acquired the transcriptional features of memory T cells, their recall ability remained compromised versus memory T cells and their chromatin landscape largely conserved as the exhausted state (79). Therefore, it suggests that exhaustion could cause persistent and stubborn epigenetic landscape alteration which is minimally reinvigorated by PD-1 blockade or removal of antigen.

Although the precise molecular mechanism maintaining the exhausted chromatin state remains unclear, multiple types of research showed that in addition to serving as a transcription factor, TOX also participates in the epigenetic remodeling in both tumors and chronic viral infection (80, 81, 87). Transcription factor NR4a has also been reported to drive Tex *via* orchestrating epigenetic changes in CD8^+^ tumor-infiltrating lymphocytes (TIL) and chimeric antigen receptor (CAR) T cells (82, 88, 89). In addition, the *de novo* methyltransferase DNMT3A was also confirmed to maintain the Tex epigenetic state (11). Ghneim et al. found that during chronic infection, activated CD8^+^ T cells whose *de novo* methylation was blocked could still retain effector functions, and even though during PD-1 blockade, the exhaustion linked *de novo* DNA methylation remained preserved. Corresponding to the differentiated degree of Tex, only the terminally differentiated Tex TILs with high PD-1 receptors acquired the exhaustion-linked DNA methylation (85) which might explain the reason that the PD-1 blockade only revives those progenitor Tex cells to some degree.

## 4 TEX in gastrointestinal cancer

Gastrointestinal cancer occurs in gastrointestinal tract and accessory organs of digestive system, including esophagus cancer, gastric cancer, colorectal cancer, and others. As gastric cancer and colorectal cancer are leading causes of cancer death globally, present review mainly focuses on these two kinds of cancer types (90, 91).

### 4.1 Gastric cancer

**Figure 3:**
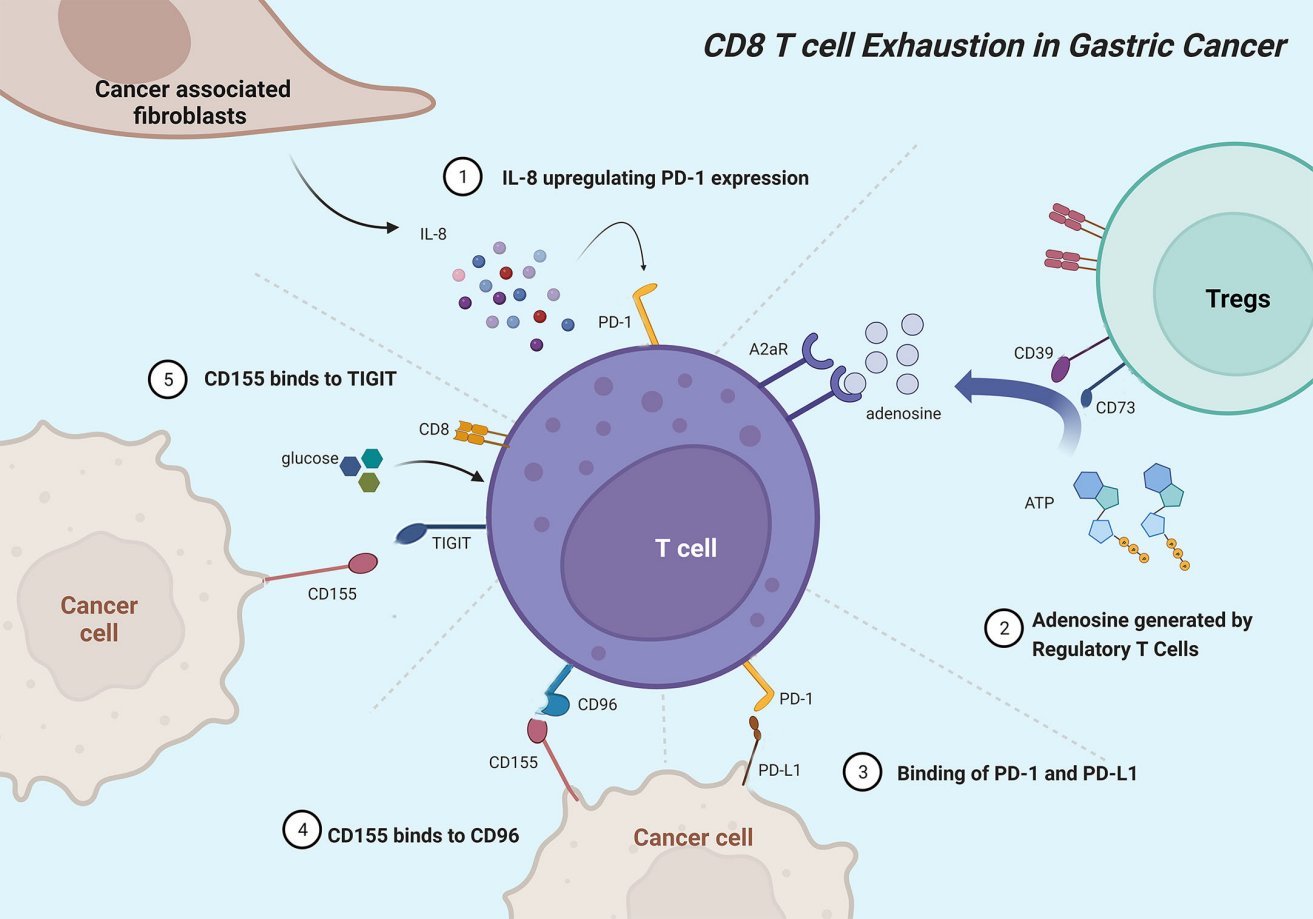
Mainly mechanisms of CD8^+^ T cell exhaustion in gastric cancer. Cancer-associated fibroblasts originate IL-8 to upregulate PD-1 expression in CD8^+^ T cells. Ectonucleotidases CD39 and CD73 catalyze adenosine that interactives with A2aR to inhibit CD8^+^ T cells. PD-L1 on tumor cells interacts with PD-1 to induce T-cell exhaustion. CD96 mainly binds to CD155 and associates with CD8^+^ T cell exhaustion phenotype. Gastric cancer cells expressing CD155 deprive CD8^+^ T cells of glucose and impair CD8^+^ T cell effector functions, which can be rescued by additional glucose.

Gastric cancer (GC) is one of the most common malignancies and the leading cause of cancer-related mortality worldwide (92). Unfortunately, though proven novel effective anticancer therapies in several cancers, programmed cell death protein 1 (PD-1) inhibitor (nivolumab) and anti-PD-L1 antibody (avelumab) showed limited benefits in improving survival outcomes for GC patients (93, 94). Hence, a clear understanding of the landscape of TEX in GC is pivotal to identifying novel targets for improving clinical management and decision-making. **Figure 3** shows the major mechanisms of T-cell exhaustion in GC.

The advancement and proliferation of single-cell RNA sequencing (scRNA-seq) technology make it possible to exactly map the status and differentiation trajectories of exhausted T cells in the tumor. Recent scRNA-seq research on tumor samples and adjacent non-tumor samples from nine untreated non-metastatic GC patients revealed that CD8^+^ T cells exhibited no significant increase of exhaustion levels and expressed low levels of exhaustion markers, including PDCD1, cytotoxic T-lymphocyte antigen 4 (CTLA-4), HAVCR2, LAG-3, and TIGIT (T-cell immunoreceptor with immunoglobulin and ITIM domains), which may partly explain the limited benefit of immunotherapy among GC patients (95). The other scRNA-seq analysis with tumor tissues, adjacent normal tissue, and matched peripheral blood of ten primary GC patients revealed that tumor-infiltrating CD8^+^ T cells reached exhausted state *via* two exhaustion trajectories. Tissue-resident memory T cells differentiate into IL-17CD8^+^ T cells (Tc17) and subsequently exhausted T cells with increasing cytolytic scores, while the blood-derived cytolytic CD8^+^ T cells turn to the exhausted population with lower exhaustion scores and decreasing cytolytic score, which are attributed to distinct transcription programs. Tc17-derived exhausted T cells highly express keratin *KRT86*, while cytolytic-cell-derived exhausted T cells express high levels of *GZMK.* As for transcription factors, *PRDM1*, *TOX2*, *NR3C1*, *CEBPD*, *ATF3*, and *EOMES* were expressed in cytolytic-cell-derived exhausted T cells, while *BHLHE40*, *CREM*, and *RUNX2* were expressed in Tc17-derived exhausted T cells (96). The high heterogeneity between the two studies may associate with differences in patient populations. The degree of confidence in the results is also limited by the limited sample sizes, which requires further validation in large-scale scRNA-seq cohorts.

Immune checkpoint molecules and regulators exert key roles in maintaining tolerance, preventing T cell hyperactivation, and regulating TEX. TIGIT, an immune checkpoint expressed on the T-cell surface, belongs to the CD28 family and binds to CD155 to suppress T-cell activation (97). In GC, TIGIT^+^CD8^+^ T cells with functional exhaustion occupy a higher proportion. GC tissue and cell lines expressing CD155 deprive CD8^+^ T cells of glucose, and impair CD8^+^ T-cell effector functions and IFNγ production, which can be rescued by additional glucose or TIGIT blockade, especially combined with PD-1 blockade (98). As a novel immune checkpoint, CD96 mainly binds to CD155 and impact CD8^+^ TEX phenotype and immunosuppressive microenvironments, leading to poor prognosis in GC (99, 100). PD-1 acts as a negative co-stimulatory receptor and induces TEX *via* interacting with programmed death ligand 1 (PD-L1) or PD-L2, which is recognized as the main mechanism of tumor cells to escape anti-tumor immune reaction (101). Elevated serum interleukin-8 (sIL-8) prominently originated from GC-associated fibroblasts upregulates PD-1 expression in CD8^+^ T cells *via* activating JAK2/STAT3 signaling and inhibiting PD-1 ubiquitination through Fbxo38 down-regulation, leading to immunosuppressive environments, lymph node metastasis, and poor prognosis (102). FoxP3^+^ Tregs in GC tissues can decompose ATP to adenosine with CD39 and CD73, and then adenosine interactives with A2aR to induce apoptosis and inhibit immune response and proliferation of CD8^+^ T cells (103, 104). Transcriptional repressor cAMP response element modulator (CREM) is induced by cAMP signaling pathway and positively correlated with exhausted marker genes in gastric adenocarcinoma (105). Dexamethasone suppress the transcription of PD-L1 and IDO1 *via* nuclear translocation of GR/STAT3 complex to inhibit TEX and immune evasion in coculture system, 3D organoid model and humanized mouse model (106).

### 4.2 Colorectal cancer

**Figure 4:**
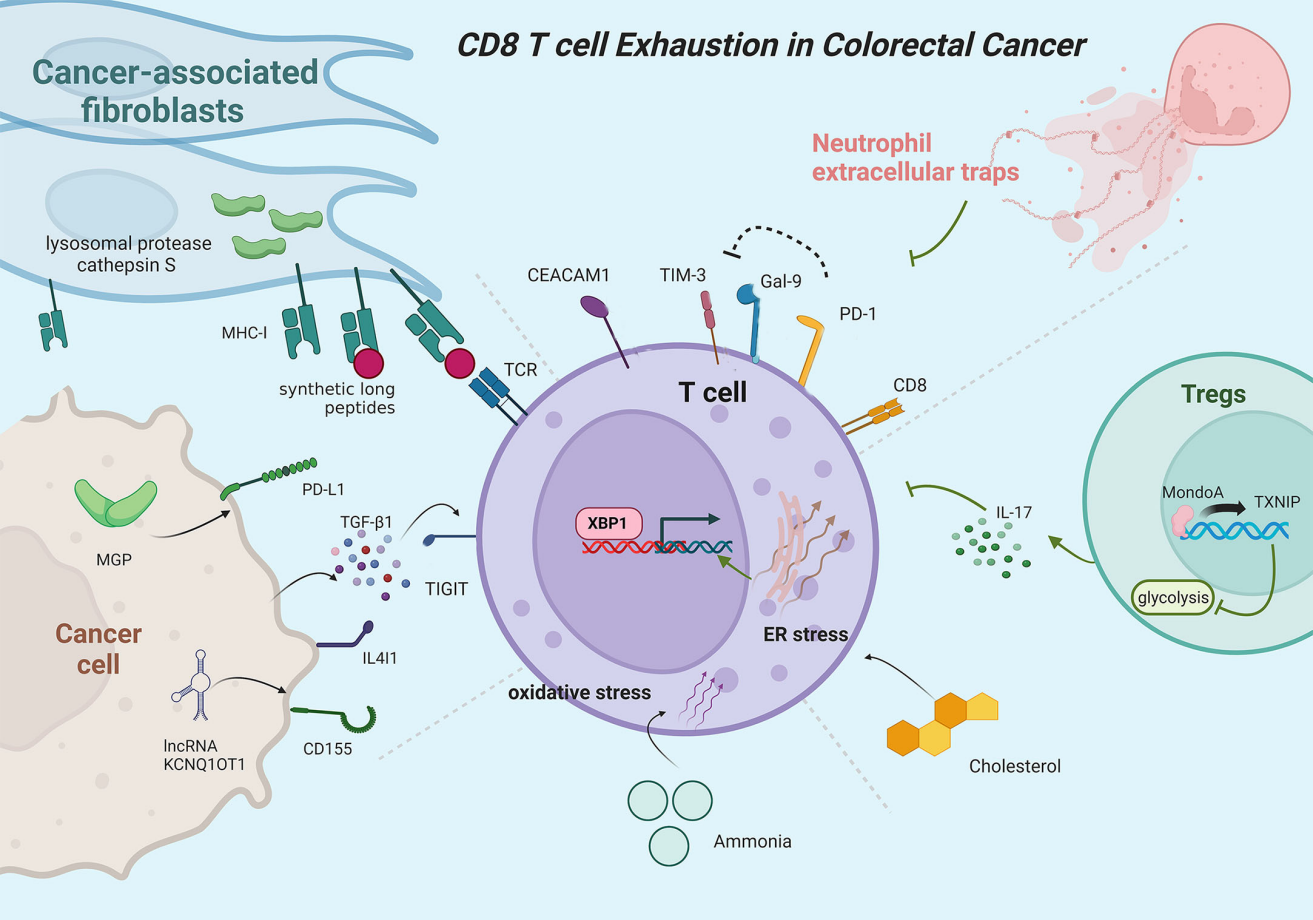
Mainly mechanisms of CD8^+^ T cell exhaustion in colorectal cancer. PD-1 protects exhausted T cells from Gal-9-induced cell death. CEACAM1 co-expresses with TIM-3 to promote T-cell exhaustion. Neutrophil extracellular traps can promote T cell exhaustion. Inhibition of the MondoA–TXNIP axis induces hyperglycolytic regulatory T cells to produce IL-17A and promotes T cell exhaustion. Cholesterol increases endoplasmic reticulum stress and activates XBP1 to induce transcription of immune checkpoints. Ammonia induces oxidative stress and T-cell exhaustion. MGP activates PD-L1 expression in cancer cells. Cancer cells can secrete TGF-β1 to inhibit T cell function. CD155 expression positively correlates with lncRNA KCNQ1OT1. Cancer-associated fibroblasts upregulate lysosomal protease cathepsin S expression and boost the capacity to cross-present synthetic long peptides, promoting T cell exhaustion.

There is growing evidence showing the pivotal roles of naïve, effector, memory, and exhausted T cells in the progression of colorectal cancer (CRC) (107). The infiltration level and exhaustion state of CD8^+^ T cells in tumor microenvironments have been recognized as two well-recognized immune escape mechanisms reflecting the resistance levels to anti-PD1 treatment (108, 109). Although MSI (microsatellite instable)/MSS (microsatellite stable) status, TMB (tumor mutational burden), POLE/POLD1 mutation, and MSI-like gene signature are widely used as indications of whether CRC a patient should receive immunotherapy, these features cannot fully explain anti-PD1 resistance and exhibit limited accuracy in predicting the response to immune checkpoint inhibitor treatment, due to they only directly or indirectly indicate the potential of high quantity of tumor-infiltrating CD8^+^ T cells in tumor samples while ignoring the exhaustion state of CD8^+^ T cells (110–112). Therefore, dissecting the TEX status in the tumor microenvironment seems to occupy a more important position in predicting the response to immunotherapy in CRC. **Figure 4** shows the major mechanisms of T cell exhaustion in CRC.

Single-cell analyses observe exhausted T cells in CRC samples but low or undetectable PD1 expression in eight polyps with CODEX imaging, suggesting that TEX only appears in CRC instead of precancerous adenomas (113). The mRNA levels of several inhibitory immune checkpoints, transcription factors, and other TEX-related markers are elevated in CRC tumor tissues, including PD-1, CTLA-4, TIGIT, T cell immunoglobulin, and mucin domain-containing protein 3 (TIM-3), CD160, CD244, killer cell lectin-like receptor subfamily G member 1 (KLRG1), Thymocyte selection-associated high-mobility-group box 2 (TOX2) and TOX3, gene name for Blimp1 (PRDM1), and Ki-67, while PD-1 and CD160 are downregulated in tumor tissue of advanced stages (114, 115). Similarly, Yassin et al. have reported that PD-1 expression increases on mucosal T cell subsets of colon and ileum with the progression of inflammation-induced CRC in the azoxymethane (AOM)/dextran sulphate sodium (DSS)-treated mice. Dysplastic aberrant crypt foci in the ascending colon increases the expression of immune checkpoint and TEX gene *PDCD1* but decreases cytotoxic T cell effector gene expression and inhibits interferon signaling (116). Yang et al. also reported a significant positive correlation between *PDCD1* and several transcription factors, including *NR4A1, BATF*, and *VDR.* Due to the persistent stimuli of tumor antigens, tumor-reactive CD103^+^CD39^+^CD8^+^ T cells in CRC tumor microenvironments display exhausted phenotypes with high expression of *CTLA4, HAVCR2, LAYN*, and *TOX (* 117). In metastatic sites of CRC, the expression levels of PD-1 and TIM-3 are abundantly upregulated compared to primary sites along with the elevated number of CD8^+^ T cells (118). The research combining single-cell mass cytometry with transcriptome sequencing in 18 patients with MSS CRC revealed that immunosenescent CD28^−^CD8^+^ T cells with decreasing proliferation dominate the T cell compartment at the immunosuppressive tumor microenvironment which takes shape early during CRC development (119). Dynamic network biomarker applied in a scRNA-seq data set identifies *CCT6A* as a biomarker for pre-exhausted T cell subpopulation, which drives *TUBA1B* expression to promote CD8^+^ TEX in CRC. Exhaustion status is also induced by the receptor-ligand interactions between terminally differentiated exhausted T cells and pre-exhausted T cells (120).

Similar to GC, higher infiltration levels of exhausted TIGIT^+^CD8^+^ T cells are observed in CRC tumor tissues with low levels of killer cytokines, including IFN-γ, IL-2, and TNF-α, but higher inhibitory receptors expression, such as PD-1, LAG-3, and TIM-3. Interestingly, CRC cells can secrete TGF-β1 to promote the expression of TIGIT, induce TIGIT^+^CD8^+^ T cell expansion, and inhibit CD8^+^ T cell function (121). The expression level of CD155 is also positively correlated with lncRNA KCNQ1OT1 in CRC cancer cells, and knockdown of lncRNA KCNQ1OT1 downregulates CD155 expression in HCT116 and SW620 cells, thereby enhancing the immune response of CD8^+^ T cells (122). Considered a novel immune checkpoint, the Natural Killer group protein 2A (NKG2A) interacts with the CD94 chain to form inhibitory receptors on NK cells and CD8^+^ T cells (123). Enriching in CRC tumor tissues, NKG2A CD8^+^ T cells characterize as tissue-resident T cells mainly in terminal exhaustion status with high functional avidity but impaired proliferative capacity (124). Galectin-9 (Gal-9) can be upregulated by interferons and interacts with TIM-3 to induce cell death of terminally exhausted T cells (PD-1TIM-3), but this process can be interfered by PD-1, which contributes to the persistence of PD-1TIM-3 T cells (125, 126). Carcinoembryonic antigen cell adhesion molecule 1 (CEACAM1) co-expresses with TIM-3 on CD8^+^ T cells to promote TEX, with TIM-3CEACAM1 CD8^+^ T cells exhibiting the most dysfunctional status and least IFN-γ production capability in CRC (127).

Besides, multiple complex mechanisms are also involved in the exhausted status of CD8^+^ T cells in CRC. MondoA, a member of Mondo family transcription factors, and thioredoxin-interacting protein (TXNIP) participate in regulating glucose metabolism and redox state (128–130). Inhibition of MondoA–TXNIP axis induces hyperglycolytic Th17-like regulatory T cells with reduced immunosuppressive functions, which formulates interleukin 17A prominent microenvironment, promotes TEX, and drives the advancement of CRC (131). Cholesterol in the tumor microenvironment increases endoplasmic reticulum stress and subsequently activates XBP1 to induce transcription of immune checkpoint in CD8^+^ T cells, contributing to TEX status with upregulated expression of PD-1, 2B4, TIM-3, and LAG-3 (132). Ammonia accumulated in the CRC microenvironment inhibits transsulfuration pathway and induce oxidative stress, paving the way to impaired T cell proliferation capacity and increased TEX. Thus, clearing tumor-associated ammonia reboots T cells and improves anti-PD-L1 efficacy (133). Elevated in CRC cancer cells, Matrix Gla protein (MGP) increases intracellular free Ca^2+^ levels, promotes NF-κB phosphorylation, and activates PD-L1 expression, leading to CD8^+^ TEX (134). PTPN2 mediates the generation of the Tim-3 subpopulation, and loss of *Ptpn2* promotes the differentiation and formation of the Tim-3^+^CD8^+^ T cells and increases Tim-3^+^CD8^+^ T cell responses in MC38 cancer models (135). In MSS CRC, vascular endothelial growth factor-A (VEGF-A) induces the expression of transcription factor TOX in T cells to enhance the exhaustion-specific transcription program and drive TEX (136). Interleukin-4-induced gene 1 (IL4I1) enhances the translocation of Aryl hydrocarbon receptor (AHR) from the cytoplasm to nuclear in CD8^+^ T cells to regulate gene expression and thereby promote TEX (137). Kynurenine upregulate immune checkpoint expression and promote TEX in CRC *via* upregulating TOX expression, while IDO1 inhibitors and TOX knockdown can restore the anti-tumor activity of CD8^+^ T cells (138). Cbl-b, an E3 ubiquitin ligase, is upregulated in PD1^+^Tim3^+^ exhausted T cells, while Cbl-b deficiency leads to reduced endogenous CD8^+^ TEX (139). Gut microbiome dysbiosis leads to elevated colon tumor susceptibility with increased CD8^+^ IFNγ T cells in precancerous colon lamina propria but decreased CD8^+^ IFNγ T cells in tumor tissues, suggesting that microbial perturbations hyper stimulate CD8^+^ T cells to promote early TEX (140). CD39^+^CD8^+^ T cells display higher PD-1 expression and partially impaired functions, which correlates with the initial progression stages of the tumor (141). Epigenetic mechanisms also participate in the regulation of T-cell exhaustion. For example, downregulated histone 3 lysine 9 trimethylation and upregulated histone 3 lysine 4 trimethylation are observed in the promoters of PD-L1 and TOX2 in CRC tumor tissues (142). High DNA methylation levels in PDCD1 promoter are related to upregulated *NR4A1* and *VDR* expression in several CD8^+^ T cells (117). Other components in the tumor microenvironment can also influence the exhaustion status of T cells. Neutrophil extracellular traps promote CD4^+^ and CD8^+^ T cells displaying functional and metabolic exhaustion with multiple inhibitory receptors (143). CRC-derived cross-presenting cancer-associated fibroblasts cognately interact with CD8^+^ T cells to suppress T cell activation, decrease cytotoxicity, and increase exhaustion marker expression, which is associated with upregulated lysosomal protease cathepsin S expression and boosted capacity to cross-present neoantigen-derived synthetic long peptides of cancer-associated fibroblasts (144).

Inflammatory bowel disease (IBD), including Crohn’s disease (CD) and ulcerative colitis (UD), causes intestinal inflammation and mucosal dysplasia, which triggers the development of IBD-related CRC (145, 146). CD39^+^ and CD39^+^PD-1^+^ CD8^+^ T cells accumulate in the intestinal tissue during inflammation in CD, and the exhaustion of CD39-expressing CD8^+^ T cells is related to attenuation and remission of disease activity, which agrees with the report that TEX informs the clinical differences in adult onset IBD (147, 148). Detailed roles of TEX in IBD deserve further exploration of the in-depth mechanisms, which may explain the likely promoting role of TEX in the course of IBD-related CRC.

With bioinformatics tools, researchers have identified multiple dysregulated molecules related to TEX and prognosis in GC or CRC tissues, such as CLDN10, C3AR1, ANTXR1, ANXA1, and PDPN (149–153). However, their exact regulatory mechanisms in T cell exhaustion remain for further validation and investigation.

## 5 Immune checkpoint inhibitors and further direction

**Figure 5:**
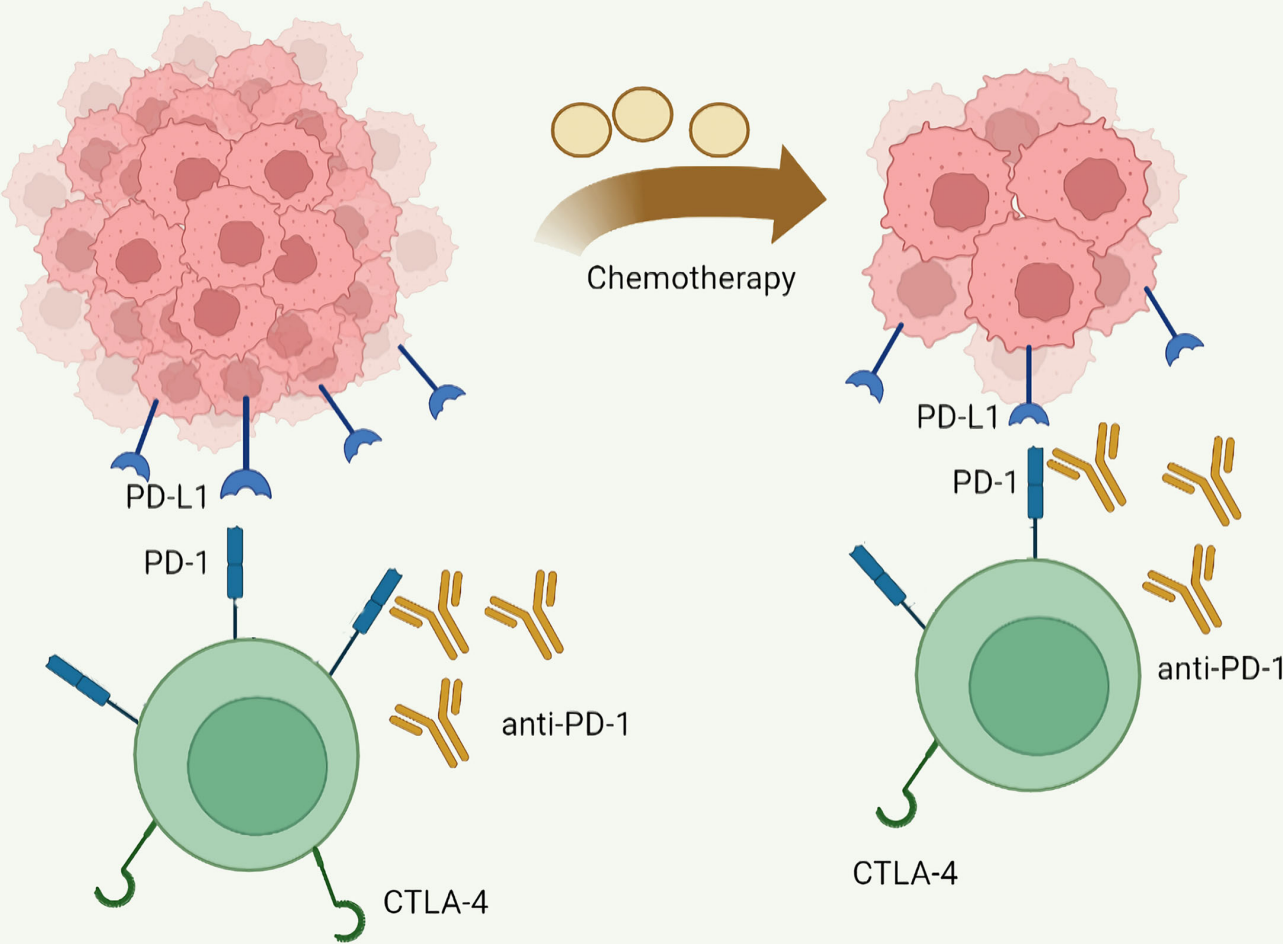
Mainly mechanisms of chemotherapy enhanced immune checkpoint inhibitor activity. Chemotherapy reduces antigen load by reducing tumor burden, thereby reducing T cell exhaustion and increasing the activity of immune checkpoint inhibitors.

PD-1, the key coinhibitory receptor on activated T cells, interacts with overexpressed PD-L1 on cancer cells, tumor-infiltrating lymphocytes, and stroma cells to inhibit T cell proliferation and function (154). CTLA-4, an inhibitory receptor primarily expressed by T cells, binds to CD80/CD86 on antigen-presenting cells resulting in impaired T cell functions (155). Since ipilimumab became the world-first approved immune checkpoint inhibitor (ICI) by the Food and Drug Administration, ICIs, mainly including monoclonal antibodies targeting PD-1, PD-L1, and CTLA-4, have been widely used and exhibited significant clinical activity in a spectrum of malignancies, including GC and CRC (156, 157). **Tables 1**, **2** list the latest representative clinical trials of ICIs in GC and CRC, respectively. A meta-analysis revealed that ICIs improve overall survival (OS) and progression free survival (PFS) for gastric/gastroesophageal junction cancer patients regardless to combined positive score (187). ICIs also exhibit high durable response rates and improved survival outcomes metastatic CRC patients with deficient mismatch repair or high microsatellite instability (188). However, although ICI is a promising method for GC and CRC, restricted effectiveness rate and common drug resistance limit its clinical application (157, 188, 189). What is worse, hyperactivated T cells induced by ICI can lead to multiple kinds of immune-related adverse events (irAEs), such as cutaneous toxicities, neurologic complications, ICI-associated myocarditis, and ICI-induced type 1 diabetes (190–195). Even so, treatment-related adverse events with ICI are less frequent and severe than chemotherapy (196). Xu et al. have summarized several biomarkers such as circulating blood cells, serum pro-inflammatory cytokines, co-expression antigens, and tumor burden as risk factors for ICIs-associated toxicities, which may help perform a timely process for ICIs-related toxicities in clinical practice (197). Moreover, ICIs combined with other interventions that are cytotoxic to tumor cells, chemotherapy for example, might enhance ICIs activity and improve clinical outcomes in GC and CRC. In a phase III clinical trial, patients with advanced GC, GEJ, or esophageal adenocarcinoma received nivolumab in combination with chemotherapy (capecitabine and oxaliplatin or leucovorin, fluorouracil, and oxaliplatin) had a longer OS, PFS, and an acceptable safety profile than did patients receiving chemotherapy alone (198). This synergy seems to be more pronounced in CRC. The efficacy of PD-1/PD-L1 inhibitors is unsatisfactory in mismatch repair proficient CRC, but patients receiving pembrolizumab combined with maraviroc exhibited prolonged disease stabilizations, limited clinical activity, improved treatment efficacy, and longer overall survival than expected (173, 199, 200). Nonetheless, the improved clinical benefits of combination therapy with ICIs and chemotherapy appear to lie in the multiple chances of response to a single agent, rather than the additive or synergistic effects of the drugs (201). Huang et al. have reported the correlation between clinical response of PD-1 blockade and the scale of reinvigorated circulating exhausted CD8^+^ T cells that are associated with the pretreatment tumor burden (202). These facts may suggest that chemotherapy reduces tumor burden to decrease antigenic load, thereby remitting TEX and improving the activity of ICIs (**Figure 5**). In a nutshell, ICI has an excellent clinical application prospect, especially combining with radiotherapy, chemotherapy, molecular target agents or other ICIs. However, considering the current limitations of ICI, novel treatment strategy and productive biomarkers for anti-tumor efficacy or toxicities of ICI need to be developed to maximize its efficacy, and deciphering the molecular mechanisms of TEX is likely to provide a rational avenue to reverse TEX and improve anti-tumor T cell activity. As described in the previous section, a variety of immune checkpoints, cytokines, and small molecule compounds are involved in the induction and persistence of TEX in GC and CRC. This suggests that targeting these molecules might alter the immunosuppressive microenvironment and activate exhausted tumor-reactive T cells, therefore, have therapeutic potential.

**Table 1:** Overview of the latest representative clinical trials of ICIs in gastric cancer.

Agent	Target	Phase	Conditions	Study design	NCT number	Ref.
Nivolumab + Andecaliximab	PD-1 / MMP9	Ib	gastric or GEJ adenocarcinoma	Andecaliximab monotherapy vs. Combination therapy of andecaliximab and nivolumab	NCT02862535	(158)
Nivolumab	PD-1	III	HER2-negative, unresectable advanced or recurrent gastric or GEJ cancer	Nivolumab plus chemotherapy versus placebo plus chemotherapy	NCT02746796	(159)
Pembrolizumab + eprenetapopt (APR-246)	PD-1/ p53	Ib	gastric or GEJ adenocarcinoma and other solid tumors	Pembrolizumab and eprenetapopt	NCT04383938	(160)
Pembrolizumab	PD-1	II	localized MSI/dMMR carcinomas suitable for curative surgery, including gastric, colon, endometrium, and other digestive cancers	Pembrolizumab	NCT04795661	(161)
Pembrolizumab	PD-1	IIb	advanced gastric or GEJ cancer	Pembrolizumab plus S-1 and oxaliplatin (SOX) or S-1 and cisplatin (SP)	NCT03382600	(162)
Pembrolizumab	PD-1	III	advanced gastric or GEJ cancer	Pembrolizumab vs. standard-dose paclitaxel	NCT02370498	(163)
Pembrolizumab	PD-1	III	advanced gastric or GEJ cancer	Pembrolizumab vs. Paclitaxel	NCT03019588	(164)
Spartalizumab	PD-1	II	resectable gastric or GEJ adenocarcinoma	Spartalizumab in combination with the FLOT regimen (fluorouracil, leucovorin, oxaliplatin, and docetaxel)	NCT04736485	(165)
Camrelizumab	PD-1	II	gastric or GEJ adenocarcinoma	Camrelizumab, apatinib, and S-1	NCT04345783	(166)
SHR-1701	PD-L1/ TGFβ	I	advanced gastric cancer and other solid tumors	SHR-1701	NCT03710265	(167)
Durvalumab + Tremelimumab	PD-L1/ CTLA-4	Ib/II	chemotherapy-refractory gastric cancer or GEJ cancer	Durvalumab plus tremelimumab or durvalumab or tremelimumab monotherapy	NCT02340975	(168)

CTLA-4, cytotoxic T lymphocyte-associated antigen-4; GEJ, gastroesophageal junction; MMP9, Matrix metalloproteinase 9; MSI/dMMR, microsatellite instability/deficient mismatch repair; PD-1, programmed death protein 1; S-1, tegafur–gimeracil–oteracil potassium.

**Table 2:** Overview of the latest representative clinical trials of ICIs in colorectal cancer.

Agent	Target	Phase	Conditions	Study design	NCT number	Ref.
Sintilimab	PD-1	Ib	DNA mismatch repair‐deficient/microsatellite instability‐high rectal cancer	Sintilimab and hypofractionated radiotherapy	NCT04636008	(169)
Dostarlimab	PD-1	II	mismatch repair-deficient stage II or III rectal adenocarcinoma	Dostarlimab followed by standard chemoradiotherapy and surgery	NCT04165772	(170)
Toripalimab	PD-1	II	microsatellite instability colorectal cancer	Toripalimab with or without the COX-2 inhibitor celecoxib	NCT03926338	(171)
Cetrelimab	PD-1	I/II	microsatellite instability-high and DNA mismatch repair-deficient colorectal cancer and other advanced solid tumors	Cetrelimab	NCT02908906	(172)
Pembrolizumab	PD-1	I	mismatch repair proficient colorectal cancer	Pembrolizumab and maraviroc followed by pembrolizumab monotherapy	NCT03274804	(173)
Pembrolizumab	PD-1	Ib	microsatellite stable colorectal cancer and other solid tumors	Ziv-aflibercept plus pembrolizumab	NCT02298959	(174)
Pembrolizumab	PD-1	II	chemotherapy-refractory metastatic colorectal cancer	Pembrolizumab combined with azacitidine	NCT02260440	(175)
Pembrolizumab	PD-1	II	advanced anal squamous cell carcinoma	Pembrolizumab	NCT02628067	(176)
Pembrolizumab	PD-1	III	microsatellite instability-high or mismatch repair-deficient metastatic colorectal cancer	Pembrolizumab vs. Chemotherapy	NCT02563002	(177)
Nivolumab	PD-1	I/Ib	mismatch repair proficient colorectal cancer	Nivolumab combined with regorafenib	NCT03712943	(178)
Toripalimab	PD-1	II	locally advanced (T3-4/N +) rectal cancer	Short-course radiotherapy combined with chemotherapy and Toripalimab	NCT04518280	(179)
Retifanlimab	PD-1	II	Locally advanced or metastatic squamous carcinoma of the anal canal	Retifanlimab	NCT03597295	(180)
Durvalumab	PD-L1	II	microsatellite stable metastatic colorectal cancer	Durvalumab and trametinib	NCT03428126	(181)
Atezolizumab	PD-L1	II	metastatic colorectal cancer	FOLFOXIRI plus bevacizumab with or without atezolizumab	NCT03721653	(182)
Avelumab	PD-L1	II	metastatic colorectal cancer	FOLFOX plus bevacizumab with or without avelumab	NCT03050814	(183)
Ipilimumab + Nivolumab	CTLA-4/PD-1	II	microsatellite instability/mismatch repair-deficient metastatic colorectal cancer	Ipilimumab plus nivolumab	NCT03350126	(184)
Tremelimumab + Durvalumab	CTLA-4/PD-L1	I	resectable colorectal cancer liver-only metastases	Tremelimumab and durvalumab preoperatively followed by durvalumab postoperatively	NCT02754856	(185)
Tremelimumab + Durvalumab	CTLA-4/PD-L1	II	colon or rectum adenocarcinoma	Tremelimumab plus durvalumab	NCT02870920	(186)

CTLA-4, cytotoxic T lymphocyte-associated antigen-4; COX, cyclooxygenases; PD-1, programmed death protein 1.

## 6 Conclusion

The functional states of CD8^+^ T cells in tumor microenvironments have received much attention due to their vital roles in the efficacy of immunotherapies. With the applications of scRNA-seq, exhausted CD8^+^ T cells are identified with multiple interconnected subpopulations with discernible heterogeneity (115). Nonetheless, the present results of scRNA-seq research might not be representative of the general population due to limited sample sizes and the high heterogeneity of patients, which requires further validation in large-scale cohorts. Researchers have also revealed numerous alters in tumor microenvironments promote the exhaustion status of T cells in GC and CRC, such as metabolite changes and cell communications. However, available research on the potential roles of TEX in IBD and IBD-related CRC is quite limited. As an inflammation-induced carcinogenesis, IBD-related CRC have different molecular pathogenesis compared with sporadic CRC that arises from adenoma (203). Thus, further exploration is needed to explore the detailed roles and mechanisms of TEX in the progression from IBD to CRC. As for anti-cancer immunotherapy, though often seen as a barrier to effective therapy, TEX is also an inevitable process that protects T cells from overstimulation-related cell death in the persistence of tumor antigens (13). Clinical trials have reported better effects of the combination of chemotherapy and inhibitory receptors blockade than monotherapy (198). Therefore, attenuating the progression of TEX seems a more promising cancer treatment modality than restoring exhausted T cells. As the current immunotherapies with ICIs are not fully satisfactory, novel treatment strategies are needed. In addition to PD-1/PD-L1 and CTLA-4, other immune checkpoints have been discovered and their functions are under investigation, which raises a fundamental question of what targets are most suited for individualizing immunotherapy among different patients. Multiple factors and cells regulating TEX in the tumor microenvironment provides a broader perspective on how to inhibit and reverse TEX and may serve as a promising avenue of synergy with anti-cancer immunotherapy.

## Author contributions

All authors have discussed the proposed scope and content of the article before drafting. J-TD, K-PY, and H-NZ wrote and revised the paper. Y-FH collected literatures. HL and ZZ reviewed and edited the manuscript. All authors contributed to the article and approved the submitted version.

## References

[1] Philip M Schietinger A. CD8(+) T cell differentiation and dysfunction in cancer. Nat Rev Immunol (2022) 22(4):209–23. doi: 10.1038/s41577-021-00574-3 PMC9792152 34253904

[2] Wherry EJ. T Cell exhaustion. Nat Immunol (2011) 12(6):492–9. doi: 10.1038/ni.2035 21739672

[3] Blank CU Haining WN Held W Hogan PG Kallies A Lugli E. Defining 'T cell exhaustion'. Nat Rev Immunol (2019) 19(11):665–74. doi: 10.1038/s41577-019-0221-9 PMC7286441 31570879

[4] Franco F Jaccard A Romero P Yu YR Ho PC. Metabolic and epigenetic regulation of T-cell exhaustion. Nat Metab (2020) 2(10):1001–12. doi: 10.1038/s42255-020-00280-9 32958939

[5] Jansen CS Prokhnevska N Master VA Sanda MG Carlisle JW Bilen MA. An intra-tumoral niche maintains and differentiates stem-like CD8 T cells. Nature. (2019) 576(7787):465–70. doi: 10.1038/s41586-019-1836-5 PMC7108171 31827286

[6] Zheng L Qin S Si W Wang A Xing B Gao R. Pan-cancer single-cell landscape of tumor-infiltrating T cells. Science (2021) 374(6574):abe6474. doi: 10.1126/science.abe6474 34914499

[7] Zheng C Zheng L Yoo JK Guo H Zhang Y Guo X. Landscape of infiltrating T cells in liver cancer revealed by single-cell sequencing. Cell. (2017) 169(7):1342–56.e16. doi: 10.1016/j.cell.2017.05.035 28622514

[8] Belk JA Daniel B Satpathy AT. Epigenetic regulation of T cell exhaustion. Nat Immunol (2022) 23(6):848–60. doi: 10.1038/s41590-022-01224-z PMC10439681 35624210

[9] Belk JA Yao W Ly N Freitas KA Chen YT Shi Q. Genome-wide CRISPR screens of T cell exhaustion identify chromatin remodeling factors that limit T cell persistence. Cancer Cell (2022) 40(7):768–86.e7. doi: 10.1016/j.ccell.2022.06.001 35750052 PMC9949532

[10] Wang Y Hu J Li Y Xiao M Wang H Tian Q. The transcription factor TCF1 preserves the effector function of exhausted CD8 T cells during chronic viral infection. Front Immunol (2019) 10:169. doi: 10.3389/fimmu.2019.00169 30814995 PMC6381939

[11] Ghoneim HE Fan Y Moustaki A Abdelsamed HA Dash P Dogra P. *De Novo* Epigenetic programs inhibit PD-1 blockade-mediated T cell rejuvenation. Cell. (2017) 170(1):142–57.e19. doi: 10.1016/j.cell.2017.06.007 28648661 PMC5568784

[12] McLane LM Abdel-Hakeem MS Wherry EJ. CD8 T cell exhaustion during chronic viral infection and cancer. Annu Rev Immunol (2019) 37:457–95. doi: 10.1146/annurev-immunol-041015-055318 30676822

[13] Chow A Perica K Klebanoff CA Wolchok JD. Clinical implications of T cell exhaustion for cancer immunotherapy. Nat Rev Clin Oncol (2022) 19(12):775–90. doi: 10.1038/s41571-022-00689-z PMC10984554 36216928

[14] Zhang N Bevan MJ. CD8(+) T cells: foot soldiers of the immune system. Immunity. (2011) 35(2):161–8. doi: 10.1016/j.immuni.2011.07.010 PMC3303224 21867926

[15] Reiser J Banerjee A. Effector, memory, and dysfunctional CD8(+) T cell fates in the antitumor immune response. J Immunol Res (2016) 2016:8941260. doi: 10.1155/2016/8941260 27314056 PMC4893440

[16] Gonzalez SM Taborda NA Rugeles MT. Role of different subpopulations of CD8(+) T cells during HIV exposure and infection. Front Immunol (2017) 8:936. doi: 10.3389/fimmu.2017.00936 28824656 PMC5545716

[17] Pu X Zhu P Zhou X He Y Wu H Du L. CD34(+) cell atlas of main organs implicates its impact on fibrosis. Cell Mol Life Sci (2022) 79(11):576. doi: 10.1007/s00018-022-04606-6 36315271 PMC11803001

[18] Wang X Waschke BC Woolaver RA Chen SMY Chen Z Wang JH. MHC class I-independent activation of virtual memory CD8 T cells induced by chemotherapeutic agent-treated cancer cells. Cell Mol Immunol (2021) 18(3):723–34. doi: 10.1038/s41423-020-0463-2 PMC8027191 32427883

[19] Oliveira G Stromhaug K Klaeger S Kula T Frederick DT Le PM. Phenotype, specificity and avidity of antitumour CD8(+) T cells in melanoma. Nature. (2021) 596(7870):119–25. doi: 10.1038/s41586-021-03704-y PMC9187974 34290406

[20] Lisci M Barton PR Randzavola LO Ma CY Marchingo JM Cantrell DA. Mitochondrial translation is required for sustained killing by cytotoxic T cells. Science (2021) 374(6565):eabe9977. doi: 10.1126/science.abe9977 34648346

[21] Wiedemann A Depoil D Faroudi M Valitutti S. Cytotoxic T lymphocytes kill multiple targets simultaneously *via* spatiotemporal uncoupling of lytic and stimulatory synapses. Proc Natl Acad Sci USA (2006) 103(29):10985–90. doi: 10.1073/pnas.0600651103 PMC1544161 16832064

[22] Magerus-Chatinet A Stolzenberg MC Loffredo MS Neven B Schaffner C Ducrot N. FAS-l, IL-10, and double-negative CD4- CD8- TCR alpha/beta+ T cells are reliable markers of autoimmune lymphoproliferative syndrome (ALPS) associated with FAS loss of function. Blood. (2009) 113(13):3027–30. doi: 10.1182/blood-2008-09-179630 19176318

[23] Caron B Patin E Rotival M Charbit B Albert ML Quintana-Murci L. Integrative genetic and immune cell analysis of plasma proteins in healthy donors identifies novel associations involving primary immune deficiency genes. Genome Med (2022) 14(1):28. doi: 10.1186/s13073-022-01032-y 35264221 PMC8905727

[24] Hellström I Hellström KE Pierce GE Yang JP. Cellular and humoral immunity to different types of human neoplasms. Nature. (1968) 220(5174):1352–4. doi: 10.1038/2201352a0 4302696

[25] Hellstrom KE Hellstrom I. From the hellstrom paradox toward cancer cure. Prog Mol Biol Transl Sci (2019) 164:1–24. doi: 10.1016/bs.pmbts.2018.11.002 31383402

[26] Ochsenbein AF Sierro S Odermatt B Pericin M Karrer U Hermans J. Roles of tumour localization, second signals and cross priming in cytotoxic T-cell induction. Nature. (2001) 411(6841):1058–64. doi: 10.1038/35082583 11429607

[27] Mascaux C Angelova M Vasaturo A Beane J Hijazi K Anthoine G. Immune evasion before tumour invasion in early lung squamous carcinogenesis. Nature. (2019) 571(7766):570–5. doi: 10.1038/s41586-019-1330-0 31243362

[28] Zinkernagel RM. Immunity against solid tumors? Int J Cancer (2001) 93(1):1–5. doi: 10.1002/ijc.1305 11391613

[29] Alavi S Emran AA Tseng HY Tiffen JC McGuire HM Hersey P. Nicotinamide inhibits T cell exhaustion and increases differentiation of CD8 effector T cells. Cancers (Basel). (2022) 14(2). doi: 10.3390/cancers14020323 PMC8774026 35053490

[30] Mitchell KG Parra ER Nelson DB Zhang J Wistuba II Fujimoto J. Tumor cellular proliferation is associated with enhanced immune checkpoint expression in stage I non-small cell lung cancer. J Thorac Cardiovasc Surg (2019) 158(3):911–9.e6. doi: 10.1016/j.jtcvs.2019.04.084 31235357 PMC8073227

[31] Yang M Lin C Wang Y Chen K Zhang H Li W. Identification of a cytokine-dominated immunosuppressive class in squamous cell lung carcinoma with implications for immunotherapy resistance. Genome Med (2022) 14(1):72. doi: 10.1186/s13073-022-01079-x 35799269 PMC9264601

[32] Beltra JC Manne S Abdel-Hakeem MS Kurachi M Giles JR Chen Z. Developmental relationships of four exhausted CD8(+) T cell subsets reveals underlying transcriptional and epigenetic landscape control mechanisms. Immunity. (2020) 52(5):825–41.e8. doi: 10.1016/j.immuni.2020.04.014 32396847 PMC8360766

[33] Kersten K Hu KH Combes AJ Samad B Harwin T Ray A. Spatiotemporal co-dependency between macrophages and exhausted CD8(+) T cells in cancer. Cancer Cell (2022) 40(6):624–38.e9. doi: 10.1016/j.ccell.2022.05.004 35623342 PMC9197962

[34] Ludwig N Whiteside TL. Potential roles of tumor-derived exosomes in angiogenesis. Expert Opin Ther Targets. (2018) 22(5):409–17. doi: 10.1080/14728222.2018.1464141 PMC6126896 29634426

[35] Whiteside TL. Exosome and mesenchymal stem cell cross-talk in the tumor microenvironment. Semin Immunol (2018) 35:69–79. doi: 10.1016/j.smim.2017.12.003 29289420 PMC5866206

[36] Lin SY Hang JF Lai CR Chan IS Shih YC Jiang LY. Loss of major histocompatibility complex class I, CD8 + tumor-infiltrating lymphocytes, and PD-L1 expression in ovarian clear cell carcinoma. Am J Surg Pathol (2023) 47(1):124–30. doi: 10.1097/PAS.0000000000001975 36221308

[37] Yoshihama S Vijayan S Sidiq T Kobayashi KS. NLRC5/CITA: a key player in cancer immune surveillance. Trends Cancer. (2017) 3(1):28–38. doi: 10.1016/j.trecan.2016.12.003 28718425 PMC5518632

[38] Dhatchinamoorthy K Colbert JD Rock KL. Cancer immune evasion through loss of MHC class I antigen presentation. Front Immunol (2021) 12:636568. doi: 10.3389/fimmu.2021.636568 33767702 PMC7986854

[39] Demel UM Böger M Yousefian S Grunert C Zhang L Hotz PW. Activated SUMOylation restricts MHC class I antigen presentation to confer immune evasion in cancer. J Clin Invest (2022) 132(9). doi: 10.1172/JCI152383 PMC9057585 35499080

[40] Xie F Zhou X Su P Li H Tu Y Du J. Breast cancer cell-derived extracellular vesicles promote CD8(+) T cell exhaustion *via* TGF-β type II receptor signaling. Nat Commun (2022) 13(1):4461. doi: 10.1038/s41467-022-31250-2 35915084 PMC9343611

[41] Ravi VM Neidert N Will P Joseph K Maier JP Kückelhaus J. T-Cell dysfunction in the glioblastoma microenvironment is mediated by myeloid cells releasing interleukin-10. Nat Commun (2022) 13(1):925. doi: 10.1038/s41467-022-28523-1 35177622 PMC8854421

[42] Qiao J Liu Z Dong C Luan Y Zhang A Moore C. Targeting tumors with IL-10 prevents dendritic cell-mediated CD8(+) T cell apoptosis. Cancer Cell (2019) 35(6):901–15.e4. doi: 10.1016/j.ccell.2019.05.005 31185213

[43] Feng Q Liu Z Yu X Huang T Chen J Wang J. Lactate increases stemness of CD8 + T cells to augment anti-tumor immunity. Nat Commun (2022) 13(1):4981. doi: 10.1038/s41467-022-32521-8 36068198 PMC9448806

[44] Elia I Rowe JH Johnson S Joshi S Notarangelo G Kurmi K. Tumor cells dictate anti-tumor immune responses by altering pyruvate utilization and succinate signaling in CD8(+) T cells. Cell Metab (2022) 34(8):1137–50.e6. doi: 10.1016/j.cmet.2022.06.008 35820416 PMC9357162

[45] Wang K Zhang Y Chen ZN. Metabolic interaction: tumor-derived lactate inhibiting CD8(+) T cell cytotoxicity in a novel route. Signal Transduct Target Ther (2023) 8(1):52. doi: 10.1038/s41392-023-01320-y 36737430 PMC9898493

[46] Giles JR Ngiow SF Manne S Baxter AE Khan O Wang P. Shared and distinct biological circuits in effector, memory and exhausted CD8(+) T cells revealed by temporal single-cell transcriptomics and epigenetics. Nat Immunol (2022) 23(11):1600–13. doi: 10.1038/s41590-022-01338-4 PMC10408358 36271148

[47] Xu J Fang Y Chen K Li S Tang S Ren Y. Single-cell RNA sequencing reveals the tissue architecture in human high-grade serous ovarian cancer. Clin Cancer Res (2022) 28(16):3590–602. doi: 10.1158/1078-0432.CCR-22-0296 PMC9662915 35675036

[48] Xu L Lu Y Deng Z Li X Shi Y Zhao K. Single-cell landscape of immunocytes in patients with extrahepatic cholangiocarcinoma. J Transl Med (2022) 20(1):210. doi: 10.1186/s12967-022-03424-5 35562760 PMC9103331

[49] Winkler F Bengsch B. Use of mass cytometry to profile human T cell exhaustion. Front Immunol (2020) 10. doi: 10.3389/fimmu.2019.03039 PMC6987473 32038613

[50] Schuyler RP Jackson C Garcia-Perez JE Baxter RM Ogolla S Rochford R. Minimizing batch effects in mass cytometry data. Front Immunol (2019) 10. doi: 10.3389/fimmu.2019.02367 PMC6803429 31681275

[51] Bengsch B Ohtani T Khan O Setty M Manne S O'Brien S. Epigenomic-guided mass cytometry profiling reveals disease-specific features of exhausted CD8 T cells. Immunity. (2018) 48(5):1029–45.e5. doi: 10.1016/j.immuni.2018.04.026 29768164 PMC6010198

[52] Ding GY Ma JQ Yun JP Chen X Ling Y Zhang S. Distribution and density of tertiary lymphoid structures predict clinical outcome in intrahepatic cholangiocarcinoma. J Hepatol (2022) 76(3):608–18. doi: 10.1016/j.jhep.2021.10.030 34793865

[53] du Bois H Heim TA Lund AW. Tumor-draining lymph nodes: At the crossroads of metastasis and immunity. Sci Immunol (2021) 6(63):eabg3551. doi: 10.1126/sciimmunol.abg3551 34516744 PMC8628268

[54] Masopust D Schenkel JM. The integration of T cell migration, differentiation and function. Nat Rev Immunol (2013) 13(5):309–20. doi: 10.1038/nri3442 23598650

[55] Kaech SM Cui W. Transcriptional control of effector and memory CD8+ T cell differentiation. Nat Rev Immunol (2012) 12(11):749–61. doi: 10.1038/nri3307 PMC4137483 23080391

[56] Obar JJ Lefrancois L. Memory CD8+ T cell differentiation. Ann N Y Acad Sci (2010) 1183:251–66. doi: 10.1111/j.1749-6632.2009.05126.x PMC2836783 20146720

[57] Wherry EJ Kurachi M. Molecular and cellular insights into T cell exhaustion. Nat Rev Immunol (2015) 15(8):486–99. doi: 10.1038/nri3862 PMC4889009 26205583

[58] Wherry EJ Barber DL Kaech SM Blattman JN Ahmed R. Antigen-independent memory CD8 T cells do not develop during chronic viral infection. Proc Natl Acad Sci USA (2004) 101(45):16004–9. doi: 10.1073/pnas.0407192101 PMC524220 15505208

[59] Shin H Blackburn SD Blattman JN Wherry EJ. Viral antigen and extensive division maintain virus-specific CD8 T cells during chronic infection. J Exp Med (2007) 204(4):941–9. doi: 10.1084/jem.20061937 PMC2118542 17420267

[60] Obar JJ Fuse S Leung EK Bellfy SC Usherwood EJ. Gammaherpesvirus persistence alters key CD8 T-cell memory characteristics and enhances antiviral protection. J Virol (2006) 80(17):8303–15. doi: 10.1128/JVI.00237-06 PMC1563881 16912282

[61] Alter G Hatzakis G Tsoukas CM Pelley K Rouleau D LeBlanc R. Longitudinal assessment of changes in HIV-specific effector activity in HIV-infected patients starting highly active antiretroviral therapy in primary infection. J Immunol (2003) 171(1):477–88. doi: 10.4049/jimmunol.171.1.477 12817033

[62] Oxenius A Price DA Gunthard HF Dawson SJ Fagard C Perrin L. Stimulation of HIV-specific cellular immunity by structured treatment interruption fails to enhance viral control in chronic HIV infection. Proc Natl Acad Sci U S A. (2002) 99(21):13747–52. doi: 10.1073/pnas.202372199 PMC129766 12370434

[63] Utzschneider DT Legat A Fuertes Marraco SA Carrie L Luescher I Speiser DE. T Cells maintain an exhausted phenotype after antigen withdrawal and population reexpansion. Nat Immunol (2013) 14(6):603–10. doi: 10.1038/ni.2606 23644506

[64] Intlekofer AM Takemoto N Wherry EJ Longworth SA Northrup JT Palanivel VR. Effector and memory CD8+ T cell fate coupled by T-bet and eomesodermin. Nat Immunol (2005) 6(12):1236–44. doi: 10.1038/ni1268 16273099

[65] Paley MA Kroy DC Odorizzi PM Johnnidis JB Dolfi DV Barnett BE. Progenitor and terminal subsets of CD8+ T cells cooperate to contain chronic viral infection. Science. (2012) 338(6111):1220–5. doi: 10.1126/science.1229620 PMC3653769 23197535

[66] Blackburn SD Shin H Freeman GJ Wherry EJ. Selective expansion of a subset of exhausted CD8 T cells by alphaPD-L1 blockade. Proc Natl Acad Sci U S A. (2008) 105(39):15016–21. doi: 10.1073/pnas.0801497105 PMC2567485 18809920

[67] McLane LM Ngiow SF Chen Z Attanasio J Manne S Ruthel G. Role of nuclear localization in the regulation and function of T-bet and eomes in exhausted CD8 T cells. Cell Rep (2021) 35(6):109120. doi: 10.1016/j.celrep.2021.109120 33979613 PMC8195461

[68] He R Hou S Liu C Zhang A Bai Q Han M. Follicular CXCR5- expressing CD8(+) T cells curtail chronic viral infection. Nature. (2016) 537(7620):412–28. doi: 10.1038/nature19317 27501245

[69] Chen Z Ji Z Ngiow SF Manne S Cai Z Huang AC. TCF-1-Centered transcriptional network drives an effector versus exhausted CD8 T cell-fate decision. Immunity. (2019) 51(5):840–55.e5. doi: 10.1016/j.immuni.2019.09.013 31606264 PMC6943829

[70] Zheng W Wei J Zebley CC Jones LL Dhungana Y Wang YD. Regnase-1 suppresses TCF-1+ precursor exhausted T-cell formation to limit CAR-t-cell responses against ALL. Blood. (2021) 138(2):122–35. doi: 10.1182/blood.2020009309 PMC8288655 33690816

[71] Wu T Ji Y Moseman EA Xu HC Manglani M Kirby M. The TCF1-Bcl6 axis counteracts type I interferon to repress exhaustion and maintain T cell stemness. Sci Immunol (2016) 1(6). doi: 10.1126/sciimmunol.aai8593 PMC5179228 28018990

[72] Im SJ Hashimoto M Gerner MY Lee J Kissick HT Burger MC. Defining CD8+ T cells that provide the proliferative burst after PD-1 therapy. Nature. (2016) 537(7620):417–21. doi: 10.1038/nature19330 PMC5297183 27501248

[73] Leong YA Chen Y Ong HS Wu D Man K Deleage C. CXCR5(+) follicular cytotoxic T cells control viral infection in b cell follicles. Nat Immunol (2016) 17(10):1187–96. doi: 10.1038/ni.3543 27487330

[74] Siddiqui I Schaeuble K Chennupati V Fuertes Marraco SA Calderon-Copete S Pais Ferreira D. Intratumoral Tcf1(+)PD-1(+)CD8(+) T cells with stem-like properties promote tumor control in response to vaccination and checkpoint blockade immunotherapy. Immunity. (2019) 50(1):195–211.e10. doi: 10.1016/j.immuni.2018.12.021 30635237

[75] Johnston RJ Poholek AC DiToro D Yusuf I Eto D Barnett B. Bcl6 and blimp-1 are reciprocal and antagonistic regulators of T follicular helper cell differentiation. Science. (2009) 325(5943):1006–10. doi: 10.1126/science.1175870 PMC2766560 19608860

[76] Nurieva RI Chung Y Martinez GJ Yang XO Tanaka S Matskevitch TD. Bcl6 mediates the development of T follicular helper cells. Science. (2009) 325(5943):1001–5. doi: 10.1126/science.1176676 PMC2857334 19628815

[77] Wherry EJ Ha SJ Kaech SM Haining WN Sarkar S Kalia V. Molecular signature of CD8+ T cell exhaustion during chronic viral infection. Immunity. (2007) 27(4):670–84. doi: 10.1016/j.immuni.2007.09.006 17950003

[78] Martinez GJ Pereira RM Aijo T Kim EY Marangoni F Pipkin ME. The transcription factor NFAT promotes exhaustion of activated CD8(+) T cells. Immunity. (2015) 42(2):265–78. doi: 10.1016/j.immuni.2015.01.006 PMC4346317 25680272

[79] Man K Gabriel SS Liao Y Gloury R Preston S Henstridge DC. Transcription factor IRF4 promotes CD8(+) T cell exhaustion and limits the development of memory-like T cells during chronic infection. Immunity. (2017) 47(6):1129–41.e5. doi: 10.1016/j.immuni.2017.11.021 29246443

[80] Scott AC Dundar F Zumbo P Chandran SS Klebanoff CA Shakiba M. TOX is a critical regulator of tumour-specific T cell differentiation. Nature. (2019) 571(7764):270–4. doi: 10.1038/s41586-019-1324-y PMC7698992 31207604

[81] Khan O Giles JR McDonald S Manne S Ngiow SF Patel KP. TOX transcriptionally and epigenetically programs CD8(+) T cell exhaustion. Nature. (2019) 571(7764):211–8. doi: 10.1038/s41586-019-1325-x PMC6713202 31207603

[82] Seo H Chen J Gonzalez-Avalos E Samaniego-Castruita D Das A Wang YH. TOX and TOX2 transcription factors cooperate with NR4A transcription factors to impose CD8(+) T cell exhaustion. Proc Natl Acad Sci U S A. (2019) 116(25):12410–5. doi: 10.1073/pnas.1905675116 PMC6589758 31152140

[83] Pauken KE Sammons MA Odorizzi PM Manne S Godec J Khan O. Epigenetic stability of exhausted T cells limits durability of reinvigoration by PD-1 blockade. Science. (2016) 354(6316):1160–5. doi: 10.1126/science.aaf2807 PMC5484795 27789795

[84] Abdel-Hakeem MS Manne S Beltra JC Stelekati E Chen Z Nzingha K. Epigenetic scarring of exhausted T cells hinders memory differentiation upon eliminating chronic antigenic stimulation. Nat Immunol (2021) 22(8):1008–19. doi: 10.1038/s41590-021-00975-5 PMC8323971 34312545

[85] Yates KB Tonnerre P Martin GE Gerdemann U Al Abosy R Comstock DE. Epigenetic scars of CD8(+) T cell exhaustion persist after cure of chronic infection in humans. Nat Immunol (2021) 22(8):1020–9. doi: 10.1038/s41590-021-00979-1 PMC8600539 34312547

[86] Tonnerre P Wolski D Subudhi S Aljabban J Hoogeveen RC Damasio M. Differentiation of exhausted CD8(+) T cells after termination of chronic antigen stimulation stops short of achieving functional T cell memory. Nat Immunol (2021) 22(8):1030–41. doi: 10.1038/s41590-021-00982-6 PMC8323980 34312544

[87] Alfei F Kanev K Hofmann M Wu M Ghoneim HE Roelli P. TOX reinforces the phenotype and longevity of exhausted T cells in chronic viral infection. Nature. (2019) 571(7764):265–9. doi: 10.1038/s41586-019-1326-9 31207605

[88] Chen J Lopez-Moyado IF Seo H Lio CJ Hempleman LJ Sekiya T. NR4A transcription factors limit CAR T cell function in solid tumours. Nature. (2019) 567(7749):530–4. doi: 10.1038/s41586-019-0985-x PMC6546093 30814732

[89] Liu X Wang Y Lu H Li J Yan X Xiao M. Genome-wide analysis identifies NR4A1 as a key mediator of T cell dysfunction. Nature. (2019) 567(7749):525–9. doi: 10.1038/s41586-019-0979-8 PMC6507425 30814730

[90] Smyth EC Nilsson M Grabsch HI van Grieken NC Lordick F. Gastric cancer. Lancet. (2020) 396(10251):635–48. doi: 10.1016/S0140-6736(20)31288-5 32861308

[91] Baidoun F Elshiwy K Elkeraie Y Merjaneh Z Khoudari G Sarmini MT. Colorectal cancer epidemiology: recent trends and impact on outcomes. Curr Drug targets. (2021) 22(9):998–1009. doi: 10.2174/1389450121999201117115717 33208072

[92] Siegel RL Miller KD Fuchs HE Jemal A. Cancer statistics, 2022. CA Cancer J Clin (2022) 72(1):7–33. doi: 10.3322/caac.21708 35020204

[93] Bang YJ Ruiz EY Van Cutsem E Lee KW Wyrwicz L Schenker M. Phase III, randomised trial of avelumab versus physician's choice of chemotherapy as third-line treatment of patients with advanced gastric or gastro-oesophageal junction cancer: primary analysis of JAVELIN gastric 300. Ann Oncol (2018) 29(10):2052–60. doi: 10.1093/annonc/mdy264 PMC6225815 30052729

[94] Kang YK Boku N Satoh T Ryu MH Chao Y Kato K. Nivolumab in patients with advanced gastric or gastro-oesophageal junction cancer refractory to, or intolerant of, at least two previous chemotherapy regimens (ONO-4538-12, ATTRACTION-2): a randomised, double-blind, placebo-controlled, phase 3 trial. Lancet. (2017) 390(10111):2461–71. doi: 10.1016/S0140-6736(17)31827-5 28993052

[95] Li Y Hu X Lin R Zhou G Zhao L Zhao D. Single-cell landscape reveals active cell subtypes and their interaction in the tumor microenvironment of gastric cancer. Theranostics. (2022) 12(8):3818–33. doi: 10.7150/thno.71833 PMC9131288 35664061

[96] Sun K Xu R Ma F Yang N Li Y Sun X. scRNA-seq of gastric tumor shows complex intercellular interaction with an alternative T cell exhaustion trajectory. Nat Commun (2022) 13(1):4943. doi: 10.1038/s41467-022-32627-z 35999201 PMC9399107

[97] Joller N Hafler JP Brynedal B Kassam N Spoerl S Levin SD. Cutting edge: TIGIT has T cell-intrinsic inhibitory functions. J Immunol (2011) 186(3):1338–42. doi: 10.4049/jimmunol.1003081 PMC3128994 21199897

[98] He W Zhang H Han F Chen X Lin R Wang W. CD155T/TIGIT signaling regulates CD8(+) T-cell metabolism and promotes tumor progression in human gastric cancer. Cancer Res (2017) 77(22):6375–88. doi: 10.1158/0008-5472.CAN-17-0381 28883004

[99] Blake SJ Dougall WC Miles JJ Teng MW Smyth MJ. Molecular pathways: targeting CD96 and TIGIT for cancer immunotherapy. Clin Cancer Res (2016) 22(21):5183–8. doi: 10.1158/1078-0432.CCR-16-0933 27620276

[100] Xu C Fang H Gu Y Yu K Wang J Lin C. Impact of intratumoural CD96 expression on clinical outcome and therapeutic benefit in gastric cancer. Cancer Sci (2022) 113(12):4070–81. doi: 10.1111/cas.15537 PMC9746045 35997524

[101] Srinivasan P Wu X Basu M Rossi C Sandler AD. PD-L1 checkpoint inhibition and anti-CTLA-4 whole tumor cell vaccination counter adaptive immune resistance: a mouse neuroblastoma model that mimics human disease. PLoS Med (2018) 15(1):e1002497. doi: 10.1371/journal.pmed.1002497 29377881 PMC5788338

[102] Li X Zhai J Shen Y Zhang T Wang Y He Y. Tumor-derived IL-8 facilitates lymph node metastasis of gastric cancer *via* PD-1 up-regulation in CD8(+) T cells. Cancer Immunol Immunother. (2022) 71(12):3057–70. doi: 10.1007/s00262-022-03223-3 PMC9588474 35633411

[103] Young A Ngiow SF Gao Y Patch AM Barkauskas DS Messaoudene M. A2AR adenosine signaling suppresses natural killer cell maturation in the tumor microenvironment. Cancer Res (2018) 78(4):1003–16. doi: 10.1158/0008-5472.CAN-17-2826 29229601

[104] Shi L Feng M Du S Wei X Song H Yixin X. Adenosine generated by regulatory T cells induces CD8(+) T cell exhaustion in gastric cancer through A2aR pathway. BioMed Res Int (2019) 2019:4093214. doi: 10.1155/2019/4093214 31930120 PMC6942766

[105] Yu K Kuang L Fu T Zhang C Zhou Y Zhu C. CREM is correlated with immune-suppressive microenvironment and predicts poor prognosis in gastric adenocarcinoma. Front Cell Dev Biol (2021) 9:697748. doi: 10.3389/fcell.2021.697748 34938728 PMC8685542

[106] Xiang Z Zhou Z Song S Li J Ji J Yan R. Dexamethasone suppresses immune evasion by inducing GR/STAT3 mediated downregulation of PD-L1 and IDO1 pathways. Oncogene. (2021) 40(31):5002–12. doi: 10.1038/s41388-021-01897-0 PMC8235907 34175886

[107] Emambux S Tachon G Junca A Tougeron D. Results and challenges of immune checkpoint inhibitors in colorectal cancer. Expert Opin Biol Ther (2018) 18(5):561–73. doi: 10.1080/14712598.2018.1445222 29471676

[108] Wu TD Madireddi S de Almeida PE Banchereau R Chen YJ Chitre AS. Peripheral T cell expansion predicts tumour infiltration and clinical response. Nature. (2020) 579(7798):274–8. doi: 10.1038/s41586-020-2056-8 32103181

[109] Sade-Feldman M Yizhak K Bjorgaard SL Ray JP de Boer CG Jenkins RW. Defining T cell states associated with response to checkpoint immunotherapy in melanoma. Cell (2019) 176(1-2):404. doi: 10.1016/j.cell.2018.12.034 30633907 PMC6647017

[110] Oliveira AF Bretes L Furtado I. Review of PD-1/PD-L1 inhibitors in metastatic dMMR/MSI-h colorectal cancer. Front Oncol (2019) 9:396. doi: 10.3389/fonc.2019.00396 31139574 PMC6527887

[111] Havel JJ Chowell D Chan TA. The evolving landscape of biomarkers for checkpoint inhibitor immunotherapy. Nat Rev Cancer. (2019) 19(3):133–50. doi: 10.1038/s41568-019-0116-x PMC6705396 30755690

[112] Li H van der Leun AM Yofe I Lubling Y Gelbard-Solodkin D van Akkooi ACJ. Dysfunctional CD8 T cells form a proliferative, dynamically regulated compartment within human melanoma. Cell. (2019) 176(4):775–89.e18. doi: 10.1016/j.cell.2018.11.043 30595452 PMC7253294

[113] Becker WR Nevins SA Chen DC Chiu R Horning AM Guha TK. Single-cell analyses define a continuum of cell state and composition changes in the malignant transformation of polyps to colorectal cancer. Nat Genet (2022) 54(7):985–95. doi: 10.1038/s41588-022-01088-x PMC9279149 35726067

[114] Saleh R Taha RZ Toor SM Sasidharan Nair V Murshed K Khawar M. Expression of immune checkpoints and T cell exhaustion markers in early and advanced stages of colorectal cancer. Cancer Immunol Immunother. (2020) 69(10):1989–99. doi: 10.1007/s00262-020-02593-w PMC7511277 32393998

[115] Thommen DS Schumacher TN. T Cell dysfunction in cancer. Cancer Cell (2018) 33(4):547–62. doi: 10.1016/j.ccell.2018.03.012 PMC7116508 29634943

[116] Ideta T Li B Flynn C Igarashi Y Lowman G Looney T. The epithelial-stromal microenvironment in early colonic neoplasia. Mol Cancer Res (2022) 20(1):56–61. doi: 10.1158/1541-7786.MCR-21-0202 34670862 PMC8738147

[117] Yang R Cheng S Luo N Gao R Yu K Kang B. Distinct epigenetic features of tumor-reactive CD8+ T cells in colorectal cancer patients revealed by genome-wide DNA methylation analysis. Genome Biol (2019) 21(1):2. doi: 10.1186/s13059-019-1921-y 31892342 PMC6937914

[118] Sakimura S Nagayama S Fukunaga M Hu Q Kitagawa A Kobayashi Y. Impaired tumor immune response in metastatic tumors is a selective pressure for neutral evolution in CRC cases. PLoS Genet (2021) 17(1):e1009113. doi: 10.1371/journal.pgen.1009113 33476333 PMC7864431

[119] Di J Liu M Fan Y Gao P Wang Z Jiang B. Phenotype molding of T cells in colorectal cancer by single-cell analysis. Int J Cancer. (2020) 146(8):2281–95. doi: 10.1002/ijc.32856 31901134

[120] Hu J Han C Zhong J Liu H Liu R Luo W. Dynamic network biomarker of pre-exhausted CD8(+) T cells contributed to T cell exhaustion in colorectal cancer. Front Immunol (2021) 12:691142. doi: 10.3389/fimmu.2021.691142 34434188 PMC8381053

[121] Liang R Zhu X Lan T Ding D Zheng Z Chen T. TIGIT promotes CD8(+)T cells exhaustion and predicts poor prognosis of colorectal cancer. Cancer Immunol Immunother. (2021) 70(10):2781–93. doi: 10.1007/s00262-021-02886-8 PMC10992182 33634371

[122] Lin ZB Long P Zhao Z Zhang YR Chu XD Zhao XX. Long noncoding RNA KCNQ1OT1 is a prognostic biomarker and mediates CD8(+) T cell exhaustion by regulating CD155 expression in colorectal cancer. Int J Biol Sci (2021) 17(7):1757–68. doi: 10.7150/ijbs.59001 PMC8120463 33994860

[123] Carretero M Cantoni C Bellon T Bottino C Biassoni R Rodriguez A. The CD94 and NKG2-a c-type lectins covalently assemble to form a natural killer cell inhibitory receptor for HLA class I molecules. Eur J Immunol (1997) 27(2):563–7. doi: 10.1002/eji.1830270230 9045931

[124] Ducoin K Oger R Bilonda Mutala L Deleine C Jouand N Desfrancois J. Targeting NKG2A to boost anti-tumor CD8 T-cell responses in human colorectal cancer. Oncoimmunology. (2022) 11(1):2046931. doi: 10.1080/2162402X.2022.2046931 35295095 PMC8920231

[125] Yang RY Rabinovich GA Liu FT. Galectins: structure, function and therapeutic potential. Expert Rev Mol Med (2008) 10:e17. doi: 10.1017/S1462399408000719 18549522

[126] Yang R Sun L Li CF Wang YH Yao J Li H. Galectin-9 interacts with PD-1 and TIM-3 to regulate T cell death and is a target for cancer immunotherapy. Nat Commun (2021) 12(1):832. doi: 10.1038/s41467-021-21099-2 33547304 PMC7864927

[127] Zhang Y Cai P Li L Shi L Chang P Liang T. Co-Expression of TIM-3 and CEACAM1 promotes T cell exhaustion in colorectal cancer patients. Int Immunopharmacol. (2017) 43:210–8. doi: 10.1016/j.intimp.2016.12.024 28038383

[128] Richards P Ourabah S Montagne J Burnol AF Postic C Guilmeau S. MondoA/ChREBP: the usual suspects of transcriptional glucose sensing; implication in pathophysiology. Metabolism. (2017) 70:133–51. doi: 10.1016/j.metabol.2017.01.033 28403938

[129] Richards P Rachdi L Oshima M Marchetti P Bugliani M Armanet M. MondoA is an essential glucose-responsive transcription factor in human pancreatic beta-cells. Diabetes. (2018) 67(3):461–72. doi: 10.2337/db17-0595 29282201

[130] Wu N Zheng B Shaywitz A Dagon Y Tower C Bellinger G. AMPK-dependent degradation of TXNIP upon energy stress leads to enhanced glucose uptake *via* GLUT1. Mol Cell (2013) 49(6):1167–75. doi: 10.1016/j.molcel.2013.01.035 PMC3615143 23453806

[131] Lu Y Li Y Liu Q Tian N Du P Zhu F. MondoA-Thioredoxin-Interacting protein axis maintains regulatory T-cell identity and function in colorectal cancer microenvironment. Gastroenterology. (2021) 161(2):575–91.e16. doi: 10.1053/j.gastro.2021.04.041 33901495

[132] Ma X Bi E Lu Y Su P Huang C Liu L. Cholesterol induces CD8(+) T cell exhaustion in the tumor microenvironment. Cell Metab (2019) 30(1):143–56.e5. doi: 10.1016/j.cmet.2019.04.002 31031094 PMC7061417

[133] Bell HN Huber AK Singhal R Korimerla N Rebernick RJ Kumar R. Microenvironmental ammonia enhances T cell exhaustion in colorectal cancer. Cell Metab (2023) 35(1):134–49.e6. doi: 10.1016/j.cmet.2022.11.013 36528023 PMC9841369

[134] Rong D Sun G Zheng Z Liu L Chen X Wu F. MGP promotes CD8(+) T cell exhaustion by activating the NF-kappaB pathway leading to liver metastasis of colorectal cancer. Int J Biol Sci (2022) 18(6):2345–61. doi: 10.7150/ijbs.70137 PMC8990480 35414780

[135] LaFleur MW Nguyen TH Coxe MA Miller BC Yates KB Gillis JE. PTPN2 regulates the generation of exhausted CD8(+) T cell subpopulations and restrains tumor immunity. Nat Immunol (2019) 20(10):1335–47. doi: 10.1038/s41590-019-0480-4 PMC6754306 31527834

[136] Kim CG Jang M Kim Y Leem G Kim KH Lee H. VEGF-a drives TOX-dependent T cell exhaustion in anti-PD-1-resistant microsatellite stable colorectal cancers. Sci Immunol (2019) 4(41). doi: 10.1126/sciimmunol.aay0555 31704735

[137] Sun H Han W Wen J Ma X. IL4I1 and tryptophan metabolites enhance AHR signals to facilitate colorectal cancer progression and immunosuppression. Am J Transl Res (2022) 14(11):7758–70. PMC9730071 36505324

[138] Wu D Zhu Y. Role of kynurenine in promoting the generation of exhausted CD8(+) T cells in colorectal cancer. Am J Transl Res (2021) 13(3):1535–47. PMC8014392 33841677

[139] Kumar J Kumar R Kumar Singh A Tsakem EL Kathania M Riese MJ. Deletion of cbl-b inhibits CD8(+) T-cell exhaustion and promotes CAR T-cell function. J Immunother Cancer (2021) 9(1). doi: 10.1136/jitc-2020-001688 PMC7813298 33462140

[140] Yu AI Zhao L Eaton KA Ho S Chen J Poe S. Gut microbiota modulate CD8 T cell responses to influence colitis-associated tumorigenesis. Cell Rep (2020) 31(1):107471. doi: 10.1016/j.celrep.2020.03.035 32268087 PMC7934571

[141] Gallerano D Ciminati S Grimaldi A Piconese S Cammarata I Focaccetti C. Genetically driven CD39 expression shapes human tumor-infiltrating CD8(+) T-cell functions. Int J Cancer. (2020) 147(9):2597–610. doi: 10.1002/ijc.33131 32483858

[142] Sasidharan Nair V Saleh R Toor SM Taha RZ Ahmed AA Kurer MA. Epigenetic regulation of immune checkpoints and T cell exhaustion markers in tumor-infiltrating T cells of colorectal cancer patients. Epigenomics. (2020) 12(21):1871–82. doi: 10.2217/epi-2020-0267 33169618

[143] Kaltenmeier C Yazdani HO Morder K Geller DA Simmons RL Tohme S. Neutrophil extracellular traps promote T cell exhaustion in the tumor microenvironment. Front Immunol (2021) 12:785222. doi: 10.3389/fimmu.2021.785222 34899751 PMC8652262

[144] Harryvan TJ Visser M de Bruin L Plug L Griffioen L Mulder A. Enhanced antigen cross-presentation in human colorectal cancer-associated fibroblasts through upregulation of the lysosomal protease cathepsin s. J Immunother Cancer (2022) 10(3). doi: 10.1136/jitc-2021-003591 PMC8915372 35264435

[145] Majumder S Shivaji UN Kasturi R Sigamani A Ghosh S Iacucci M. Inflammatory bowel disease-related colorectal cancer: past, present and future perspectives. World J Gastrointest Oncol (2022) 14(3):547–67. doi: 10.4251/wjgo.v14.i3.547 PMC8919014 35321275

[146] Ephraim R Feehan J Fraser S Nurgali K Apostolopoulos V. Cancer immunotherapy: the checkpoint between chronic colitis and colorectal cancer. Cancers (Basel). (2022) 14(24). doi: 10.3390/cancers14246131 PMC9776998 36551617

[147] Globig AM Mayer LS Heeg M Andrieux G Ku M Otto-Mora P. Exhaustion of CD39-expressing CD8(+) T cells in crohn's disease is linked to clinical outcome. Gastroenterology. (2022) 163(4):965–81.e31. doi: 10.1053/j.gastro.2022.06.045 35738329

[148] McKinney EF Lee JC Jayne DR Lyons PA Smith KG. T-Cell exhaustion, co-stimulation and clinical outcome in autoimmunity and infection. Nature. (2015) 523(7562):612–6. doi: 10.1038/nature14468 PMC4623162 26123020

[149] Rao X Jiang J Liang Z Zhang J Zhuang Z Qiu H. Down-regulated CLDN10 predicts favorable prognosis and correlates with immune infiltration in gastric cancer. Front Genet (2021) 12:747581. doi: 10.3389/fgene.2021.747581 34721537 PMC8548647

[150] Yang H Li L Liu X Zhao Y. High expression of the component 3a receptor 1 (C3AR1) gene in stomach adenocarcinomas infers a poor prognosis and high immune-infiltration levels. Med Sci Monit (2021) 27:e927977. doi: 10.12659/MSM.927977 33539329 PMC7871482

[151] Huang X Zhang J Zheng Y. ANTXR1 is a prognostic biomarker and correlates with stromal and immune cell infiltration in gastric cancer. Front Mol Biosci (2020) 7:598221. doi: 10.3389/fmolb.2020.598221 33385012 PMC7770144

[152] Liang Z Li X. Identification of ANXA1 as a potential prognostic biomarker and correlating with immune infiltrates in colorectal cancer. Autoimmunity. (2021) 54(2):76–87. doi: 10.1080/08916934.2021.1887148 33596760

[153] Hu L Zhang P Sun W Zhou L Chu Q Chen Y. PDPN is a prognostic biomarker and correlated with immune infiltrating in gastric cancer. Med (Baltimore). (2020) 99(19):e19957. doi: 10.1097/MD.0000000000019957 PMC7220208 32384443

[154] Tang W Pan X Han D Rong D Zhang M Yang L. Clinical significance of CD8(+) T cell immunoreceptor with ig and ITIM domains(+) in locally advanced gastric cancer treated with SOX regimen after D2 gastrectomy. Oncoimmunology. (2019) 8(6):e1593807. doi: 10.1080/2162402X.2019.1593807 31069158 PMC6493216

[155] Van Coillie S Wiernicki B Xu J. Molecular and cellular functions of CTLA-4. Adv Exp Med Biol (2020) 1248:7–32. doi: 10.1007/978-981-15-3266-5_2 32185705

[156] Robert C Thomas L Bondarenko I O'Day S Weber J Garbe C. Ipilimumab plus dacarbazine for previously untreated metastatic melanoma. N Engl J Med (2011) 364(26):2517–26. doi: 10.1056/NEJMoa1104621 21639810

[157] Bagchi S Yuan R Engleman EG. Immune checkpoint inhibitors for the treatment of cancer: clinical impact and mechanisms of response and resistance. Annu Rev Pathol (2021) 16:223–49. doi: 10.1146/annurev-pathol-042020-042741 33197221

[158] Yoshikawa AK Yamaguchi K Muro K Takashima A Ichimura T Sakai D. Safety and tolerability of andecaliximab as monotherapy and in combination with an anti-PD-1 antibody in Japanese patients with gastric or gastroesophageal junction adenocarcinoma: a phase 1b study. J Immunother Cancer (2022) 10(1). doi: 10.1136/jitc-2021-003518 PMC8739432 34992093

[159] Kang YK Chen LT Ryu MH Oh DY Oh SC Chung HC. Nivolumab plus chemotherapy versus placebo plus chemotherapy in patients with HER2-negative, untreated, unresectable advanced or recurrent gastric or gastro-oesophageal junction cancer (ATTRACTION-4): a randomised, multicentre, double-blind, placebo-controlled, phase 3 trial. Lancet Oncol (2022) 23(2):234–47. doi: 10.1016/S1470-2045(21)00692-6 35030335

[160] Park H Shapiro GI Gao X Mahipal A Starr J Furqan M. Phase ib study of eprenetapopt (APR-246) in combination with pembrolizumab in patients with advanced or metastatic solid tumors. ESMO Open (2022) 7(5):100573. doi: 10.1016/j.esmoop.2022.100573 36084396 PMC9588880

[161] Coutzac C Bibeau F Ben Abdelghani M Aparicio T Cohen R Coquan E. Immunotherapy in MSI/dMMR tumors in the perioperative setting: the IMHOTEP trial. Dig Liver Dis (2022) 54(10):1335–41. doi: 10.1016/j.dld.2022.07.008 35907691

[162] Yamaguchi K Minashi K Sakai D Nishina T Omuro Y Tsuda M. Phase IIb study of pembrolizumab combined with s-1 + oxaliplatin or s-1 + cisplatin as first-line chemotherapy for gastric cancer. Cancer Sci (2022) 113(8):2814–27. doi: 10.1111/cas.15462 PMC9357620 35701865

[163] Fuchs CS Ozguroglu M Bang YJ Di Bartolomeo M Mandala M Ryu MH. Pembrolizumab versus paclitaxel for previously treated PD-L1-positive advanced gastric or gastroesophageal junction cancer: 2-year update of the randomized phase 3 KEYNOTE-061 trial. Gastric Cancer. (2022) 25(1):197–206. doi: 10.1007/s10120-021-01227-z 34468869 PMC8732941

[164] Chung HC Kang YK Chen Z Bai Y Wan Ishak WZ Shim BY. Pembrolizumab versus paclitaxel for previously treated advanced gastric or gastroesophageal junction cancer (KEYNOTE-063): a randomized, open-label, phase 3 trial in Asian patients. Cancer. (2022) 128(5):995–1003. doi: 10.1002/cncr.34019 34878659 PMC9299889

[165] Dos Santos M Lequesne J Leconte A Corbinais S Parzy A Guilloit JM. Perioperative treatment in resectable gastric cancer with spartalizumab in combination with fluorouracil, leucovorin, oxaliplatin and docetaxel (FLOT): a phase II study (GASPAR). BMC Cancer. (2022) 22(1):537. doi: 10.1186/s12885-022-09623-z 35549674 PMC9097175

[166] Jing C Wang J Zhu M Bai Z Zhao B Zhang J. Camrelizumab combined with apatinib and s-1 as second-line treatment for patients with advanced gastric or gastroesophageal junction adenocarcinoma: a phase 2, single-arm, prospective study. Cancer Immunol Immunother. (2022) 71(11):2597–608. doi: 10.1007/s00262-022-03174-9 PMC8932366 35304622

[167] Liu D Zhou J Wang Y Li M Jiang H Liu Y. Bifunctional anti-PD-L1/TGF-betaRII agent SHR-1701 in advanced solid tumors: a dose-escalation, dose-expansion, and clinical-expansion phase 1 trial. BMC Med (2022) 20(1):408. doi: 10.1186/s12916-022-02605-9 36280870 PMC9594927

[168] Kelly RJ Lee J Bang YJ Almhanna K Blum-Murphy M Catenacci DVT. Safety and efficacy of durvalumab and tremelimumab alone or in combination in patients with advanced gastric and gastroesophageal junction adenocarcinoma. Clin Cancer Res (2020) 26(4):846–54. doi: 10.1158/1078-0432.CCR-19-2443 PMC7748730 31676670

[169] Li X Fang C Wang X Yu Y Wang Z Qiu M. Neoadjuvant treatment of sintilimab plus hypofractionated radiotherapy for MSI-H/dMMR rectal cancer: a prospective, multicenter, phase ib study. Cancer Med (2022) 11(23):4405–10. doi: 10.1002/cam4.4720 PMC9741977 35352512

[170] Cercek A Lumish M Sinopoli J Weiss J Shia J Lamendola-Essel M. PD-1 blockade in mismatch repair-deficient, locally advanced rectal cancer. N Engl J Med (2022) 386(25):2363–76. doi: 10.1056/NEJMoa2201445 PMC9492301 35660797

[171] Hu H Kang L Zhang J Wu Z Wang H Huang M. Neoadjuvant PD-1 blockade with toripalimab, with or without celecoxib, in mismatch repair-deficient or microsatellite instability-high, locally advanced, colorectal cancer (PICC): a single-centre, parallel-group, non-comparative, randomised, phase 2 trial. Lancet Gastroenterol Hepatol (2022) 7(1):38–48. doi: 10.1016/S2468-1253(21)00348-4 34688374

[172] Felip E Moreno V Morgensztern D Curigliano G Rutkowski P Trigo JM. First-in-human, open-label, phase 1/2 study of the monoclonal antibody programmed cell death protein-1 (PD-1) inhibitor cetrelimab (JNJ-63723283) in patients with advanced cancers. Cancer Chemother Pharmacol (2022) 89(4):499–514. doi: 10.1007/s00280-022-04414-6 35298698 PMC8956549

[173] Haag GM Springfeld C Grun B Apostolidis L Zschabitz S Dietrich M. Pembrolizumab and maraviroc in refractory mismatch repair proficient/microsatellite-stable metastatic colorectal cancer - the PICCASSO phase I trial. Eur J Cancer. (2022) 167:112–22. doi: 10.1016/j.ejca.2022.03.017 35427833

[174] Rahma OE Tyan K Giobbie-Hurder A Brohl AS Bedard PL Renouf DJ. Phase IB study of ziv-aflibercept plus pembrolizumab in patients with advanced solid tumors. J Immunother Cancer (2022) 10(3). doi: 10.1136/jitc-2021-003569 PMC8915279 35264434

[175] Kuang C Park Y Augustin RC Lin Y Hartman DJ Seigh L. Pembrolizumab plus azacitidine in patients with chemotherapy refractory metastatic colorectal cancer: a single-arm phase 2 trial and correlative biomarker analysis. Clin Epigenetics. (2022) 14(1):3. doi: 10.1186/s13148-021-01226-y 34991708 PMC8740438

[176] Marabelle A Cassier PA Fakih M Kao S Nielsen D Italiano A. Pembrolizumab for previously treated advanced anal squamous cell carcinoma: results from the non-randomised, multicohort, multicentre, phase 2 KEYNOTE-158 study. Lancet Gastroenterol Hepatol (2022) 7(5):446–54. doi: 10.1016/S2468-1253(21)00382-4 PMC12012850 35114169

[177] Diaz LA Jr. Shiu KK Kim TW Jensen BV Jensen LH Punt C. Pembrolizumab versus chemotherapy for microsatellite instability-high or mismatch repair-deficient metastatic colorectal cancer (KEYNOTE-177): final analysis of a randomised, open-label, phase 3 study. Lancet Oncol (2022) 23(5):659–70. doi: 10.1016/S1470-2045(22)00197-8 PMC9533375 35427471

[178] Kim RD Kovari BP Martinez M Xie H Sahin IH Mehta R. A phase I/Ib study of regorafenib and nivolumab in mismatch repair proficient advanced refractory colorectal cancer. Eur J Cancer. (2022) 169:93–102. doi: 10.1016/j.ejca.2022.03.026 35526308

[179] Wang Y Shen L Wan J Zhang H Wu R Wang J. Short-course radiotherapy combined with CAPOX and toripalimab for the total neoadjuvant therapy of locally advanced rectal cancer: a randomized, prospective, multicentre, double-arm, phase II trial (TORCH). BMC Cancer. (2022) 22(1):274. doi: 10.1186/s12885-022-09348-z 35291966 PMC8922781

[180] Rao S Anandappa G Capdevila J Dahan L Evesque L Kim S. A phase II study of retifanlimab (INCMGA00012) in patients with squamous carcinoma of the anal canal who have progressed following platinum-based chemotherapy (POD1UM-202). ESMO Open (2022) 7(4):100529. doi: 10.1016/j.esmoop.2022.100529 35816951 PMC9463376

[181] Johnson B Haymaker CL Parra ER Soto LMS Wang X Thomas JV. Phase II study of durvalumab (anti-PD-L1) and trametinib (MEKi) in microsatellite stable (MSS) metastatic colorectal cancer (mCRC). J Immunother Cancer (2022) 10(8). doi: 10.1136/jitc-2022-005332 PMC9422817 36007963

[182] Antoniotti C Rossini D Pietrantonio F Catteau A Salvatore L Lonardi S. Upfront FOLFOXIRI plus bevacizumab with or without atezolizumab in the treatment of patients with metastatic colorectal cancer (AtezoTRIBE): a multicentre, open-label, randomised, controlled, phase 2 trial. Lancet Oncol (2022) 23(7):876–87. doi: 10.1016/S1470-2045(22)00274-1 35636444

[183] Redman JM Tsai YT Weinberg BA Donahue RN Gandhy S Gatti-Mays ME. A randomized phase II trial of mFOLFOX6 + bevacizumab alone or with AdCEA vaccine + avelumab immunotherapy for untreated metastatic colorectal cancer. Oncologist. (2022) 27(3):198–209. doi: 10.1093/oncolo/oyab046 35274710 PMC8914498

[184] Cohen R Bennouna J Meurisse A Tournigand C de la Fouchardiere C Tougeron D. RECIST and iRECIST criteria for the evaluation of nivolumab plus ipilimumab in patients with microsatellite instability-high/mismatch repair-deficient metastatic colorectal cancer: the GERCOR NIPICOL phase II study. J Immunother Cancer (2020) 8(2). doi: 10.1136/jitc-2020-001499 PMC7640587 33148693

[185] Kanikarla Marie P Haymaker C Parra ER Kim YU Lazcano R Gite S. Pilot clinical trial of perioperative durvalumab and tremelimumab in the treatment of resectable colorectal cancer liver metastases. Clin Cancer Res (2021) 27(11):3039–49. doi: 10.1158/1078-0432.CCR-21-0163 PMC8172528 33811152

[186] Chen EX Jonker DJ Loree JM Kennecke HF Berry SR Couture F. Effect of combined immune checkpoint inhibition vs best supportive care alone in patients with advanced colorectal cancer: the Canadian cancer trials group CO.26 study. JAMA Oncol (2020) 6(6):831–8. doi: 10.1001/jamaoncol.2020.0910 PMC7206536 32379280

[187] Dubois M Liscia N Brunetti O Ziranu P Lai E Argentiero A. The role of immune checkpoint inhibitors in the treatment sequence of advanced gastric or gastro-esophageal junction cancer: a systematic review and meta-analysis of randomized trials. Crit Rev oncology/hematology. (2022) 173:103674. doi: 10.1016/j.critrevonc.2022.103674 35364261

[188] Borelli B Antoniotti C Carullo M Germani MM Conca V Masi G. Immune-checkpoint inhibitors (ICIs) in metastatic colorectal cancer (mCRC) patients beyond microsatellite instability. Cancers (Basel). (2022) 14(20). doi: 10.3390/cancers14204974 PMC9599678 36291761

[189] Kono K Nakajima S Mimura K. Current status of immune checkpoint inhibitors for gastric cancer. Gastric Cancer. (2020) 23(4):565–78. doi: 10.1007/s10120-020-01090-4 32468420

[190] Wang M Zhai X Li J Guan J Xu S Li Y. The role of cytokines in predicting the response and adverse events related to immune checkpoint inhibitors. Front Immunol (2021) 12:670391. doi: 10.3389/fimmu.2021.670391 34367136 PMC8339552

[191] Yoshikawa Y Imamura M Yamauchi M Hayes CN Aikata H Okamoto W. Prevalence of immune-related adverse events and anti-tumor efficacy following immune checkpoint inhibitor therapy in Japanese patients with various solid tumors. BMC Cancer. (2022) 22(1):1232. doi: 10.1186/s12885-022-10327-7 36447159 PMC9706984

[192] Ding JT Yang KP Lin KL Cao YK Zou F. Mechanisms and therapeutic strategies of immune checkpoint molecules and regulators in type 1 diabetes. Front Endocrinol (Lausanne). (2022) 13:1090842. doi: 10.3389/fendo.2022.1090842 36704045 PMC9871554

[193] Haugh AM Probasco JC Johnson DB. Neurologic complications of immune checkpoint inhibitors. Expert Opin Drug Saf. (2020) 19(4):479–88. doi: 10.1080/14740338.2020.1738382 PMC7192781 32126176

[194] Quach HT Johnson DB LeBoeuf NR Zwerner JP Dewan AK. Cutaneous adverse events caused by immune checkpoint inhibitors. J Am Acad Dermatol (2021) 85(4):956–66. doi: 10.1016/j.jaad.2020.09.054 34332798

[195] Moslehi J Lichtman AH Sharpe AH Galluzzi L Kitsis RN. Immune checkpoint inhibitor-associated myocarditis: manifestations and mechanisms. J Clin Invest. (2021) 131(5). doi: 10.1172/JCI145186 PMC7919710 33645548

[196] Chen C Zhang F Zhou N Gu YM Zhang YT He YD. Efficacy and safety of immune checkpoint inhibitors in advanced gastric or gastroesophageal junction cancer: a systematic review and meta-analysis. Oncoimmunology. (2019) 8(5):e1581547. doi: 10.1080/2162402X.2019.1581547 31069144 PMC6492970

[197] Xu Y Fu Y Zhu B Wang J Zhang B. Predictive biomarkers of immune checkpoint inhibitors-related toxicities. Front Immunol (2020) 11:2023. doi: 10.3389/fimmu.2020.02023 33123120 PMC7572846

[198] Janjigian YY Shitara K Moehler M Garrido M Salman P Shen L. First-line nivolumab plus chemotherapy versus chemotherapy alone for advanced gastric, gastro-oesophageal junction, and oesophageal adenocarcinoma (CheckMate 649): a randomised, open-label, phase 3 trial. Lancet. (2021) 398(10294):27–40. doi: 10.1016/S0140-6736(21)00797-2 34102137 PMC8436782

[199] Topalian SL Hodi FS Brahmer JR Gettinger SN Smith DC McDermott DF. Safety, activity, and immune correlates of anti-PD-1 antibody in cancer. N Engl J Med (2012) 366(26):2443–54. doi: 10.1056/NEJMoa1200690 PMC3544539 22658127

[200] Parikh AR Szabolcs A Allen JN Clark JW Wo JY Raabe M. Radiation therapy enhances immunotherapy response in microsatellite stable colorectal and pancreatic adenocarcinoma in a phase II trial. Nat Cancer. (2021) 2(11):1124–35. doi: 10.1038/s43018-021-00269-7 PMC8809884 35122060

[201] Palmer AC Izar B Hwangbo H Sorger PK. Predictable clinical benefits without evidence of synergy in trials of combination therapies with immune-checkpoint inhibitors. Clin Cancer Res (2022) 28(2):368–77. doi: 10.1158/1078-0432.CCR-21-2275 PMC9068233 35045958

[202] Huang AC Postow MA Orlowski RJ Mick R Bengsch B Manne S. T-Cell invigoration to tumour burden ratio associated with anti-PD-1 response. Nature. (2017) 545(7652):60–5. doi: 10.1038/nature22079 PMC5554367 28397821

[203] Shah SC Itzkowitz SH. Colorectal cancer in inflammatory bowel disease: mechanisms and management. Gastroenterology. (2022) 162(3):715–30.e3. doi: 10.1053/j.gastro.2021.10.035 34757143 PMC9003896

